# Temporally coordinated expression of nuclear genes encoding chloroplast proteins in wheat promotes *Puccinia striiformis* f. sp. *tritici* infection

**DOI:** 10.1038/s42003-022-03780-4

**Published:** 2022-08-22

**Authors:** Pilar Corredor-Moreno, Roshani Badgami, Sally Jones, Diane G. O. Saunders

**Affiliations:** John Innes Centre, Norwich Research Park, Norwich, UK

**Keywords:** Plant sciences, Plant breeding, Microbe

## Abstract

Targeting host processes that allow pathogens to thrive can be invaluable in resistance breeding. Here, we generated a deep-sequencing transcriptome time course for *Puccinia striiformis* f. sp. *tritici* (*Pst*) infection on wheat and compared datasets from three wheat varieties with different levels of susceptibility to two tested pathogen isolates. We sought genes specifically altered in a susceptible host as candidates that might support colonisation. Host responses differed between *Pst*-varietal pairs most prominently early during infection. Notably, however, nuclear genes encoding chloroplast-localised proteins (NGCPs) exhibited temporal coordination of expression profiles that differed at later time points in relation to *Pst* susceptibility. Disrupting one such NGCP, encoding the chloroplast-localised RNA binding protein TaCSP41a, led to lower *Pst* susceptibility. These analyses thus highlight NGCPs as prime targets for *Pst* manipulation during infection and point to *TaCSP41a* disruption as a potential source of *Pst* resistance for breeding programmes.

## Abstract (web summary)

A transcriptome time course of *Puccinia striiformis* f. sp. *tritici* (*Pst*) infection reveals nuclear genes encoding chloroplast-localized proteins are manipulated during infection and highlights *TaCSP41a* disruption as a target for resistance breeding.

## Introduction

Plants have highly sensitive and specific recognition systems that thwart colonisation by most potential phytopathogens. Some pathogens, however, overcome plant structural barriers and defence responses and reprogramme the host plant circuitry to support their own growth and development^1^. Within the target plant species, particular varieties may be able to recognise certain pathogen strains (races) and mount an effective defence response, thereby halting pathogen proliferation^2^. Such race-specific resistance involves recognition of pathogen effector proteins known as avirulence (Avr) proteins (secreted by the pathogen into the host plant) by corresponding host plant resistance (R) proteins, leading to localised cell death that halts the progress of infection^3^. Deletion or modification of the Avr effectors can circumvent recognition by the host plant, resulting in the emergence of novel pathogen races and limiting the utility of *R* genes for plant breeding^4^.

An alternative approach to engineering pathogen resistance entails the identification and disruption of host processes that are actively manipulated by the pathogen to support its ingress^5^. Most pathogens—especially biotrophic pathogens—rely on the cooperation of the host plant to proliferate. Pathogens develop an intimate association with their host plant, secreting effector proteins that induce structural changes to support infection stage transitions and that manipulate biosynthetic pathways and host metabolism to facilitate nutrient acquisition^6^. Consequently, mutation or deletion of host targets with essential roles in supporting pathogen colonisation typically leads to a recessive loss of susceptibility^5^. Unlike *R* genes, resistance conferred by disrupting the host genes required to support pathogen ingress can provide broad-spectrum, non-race-specific resistance. One long-standing example is the enhanced resistance against powdery mildew in barley (*Hordeum vulgare*) due to loss of function mutations at *Mildew-resistance locus* (*Mlo*)^7^. The broad, non-race-specific resistance conferred by loss of *Mlo* function has led to its widespread incorporation in breeding pipelines^8^. Thus, the identification of host genes required to support pathogen progression can provide a valuable source of resistance for breeding programmes.

To elucidate the complexities and specific stages of host responses during pathogen colonisation, large-scale transcriptome deep-sequencing (RNA-seq) has proved a powerful approach^9^. Here, we set out to identify such host processes in wheat (*Triticum aestivum*) during infection with the genetically intractable and obligate biotrophic fungal pathogen *Puccinia striiformis* f. sp. *tritici* (*Pst*), the causal agent of wheat yellow (stripe) rust. This devastating disease is now the most damaging of the three wheat rusts (stem rust [black], leaf rust [brown], and yellow rust) worldwide due to its recent geographic expansion^10^. *Pst* causes yield losses by diverting nutrients normally earmarked for grain development, resulting in wrinkled grain with inferior nutritional value^11^. In Europe, exotic incursions of new *Pst* races over the past two decades have rendered many previously resistant wheat varieties vulnerable to infection^12,13^, which underscores the urgency in identifying sources of yellow rust resistance for plant breeding.

We took advantage of host plant varietal variation to characterise the spectrum from fully susceptible to resistant interactions during *Pst* infection, with the goal of identifying candidate targets for resistance breeding programmes. We generated a detailed time course over the full *Pst* infection cycle, using three European commercial wheat varieties, Oakley, Solstice, and Santiago, which display different levels of susceptibility to two tested *Pst* isolates^14^. The *Pst* isolates 13/14 and F22 were selected as representative of the two currently dominant *Pst* lineages in Europe, Warrior (*PstS7*) and Warrior- (*PstS10*)^15^. The resulting transcriptional profiles revealed marked differences between susceptible and resistant responses, starting early during infection at one-day post-inoculation (dpi), with gene expression profiles clustering according to the *Pst* isolate and wheat variety. We also uncovered temporally coordinated regulation in the expression of nuclear genes encoding chloroplast-localised proteins (NGCPs) that differ later during infection as a function of *Pst* susceptibility. From these candidates, we selected the NGCP TaCSP41a, an RNA binding protein, for further investigation during *Pst* infection. *TaCSP41a-*A-disruption mutants showed significantly reduced susceptibility to *Pst* isolate 13/14, indicating a role for TaCSP41a in supporting *Pst* disease progression. Overall, our analyses emphasize that NGCPs are targets for *Pst* manipulation during infection, provide candidates for further investigation and indicate that *TaCSP41a* disruption may be a source of *Pst* resistance worthy of further exploration in breeding.

## Results

### RNA-seq read mapping in wheat and *Pst* reflects the susceptibility of the interaction

**Fig. 1:**
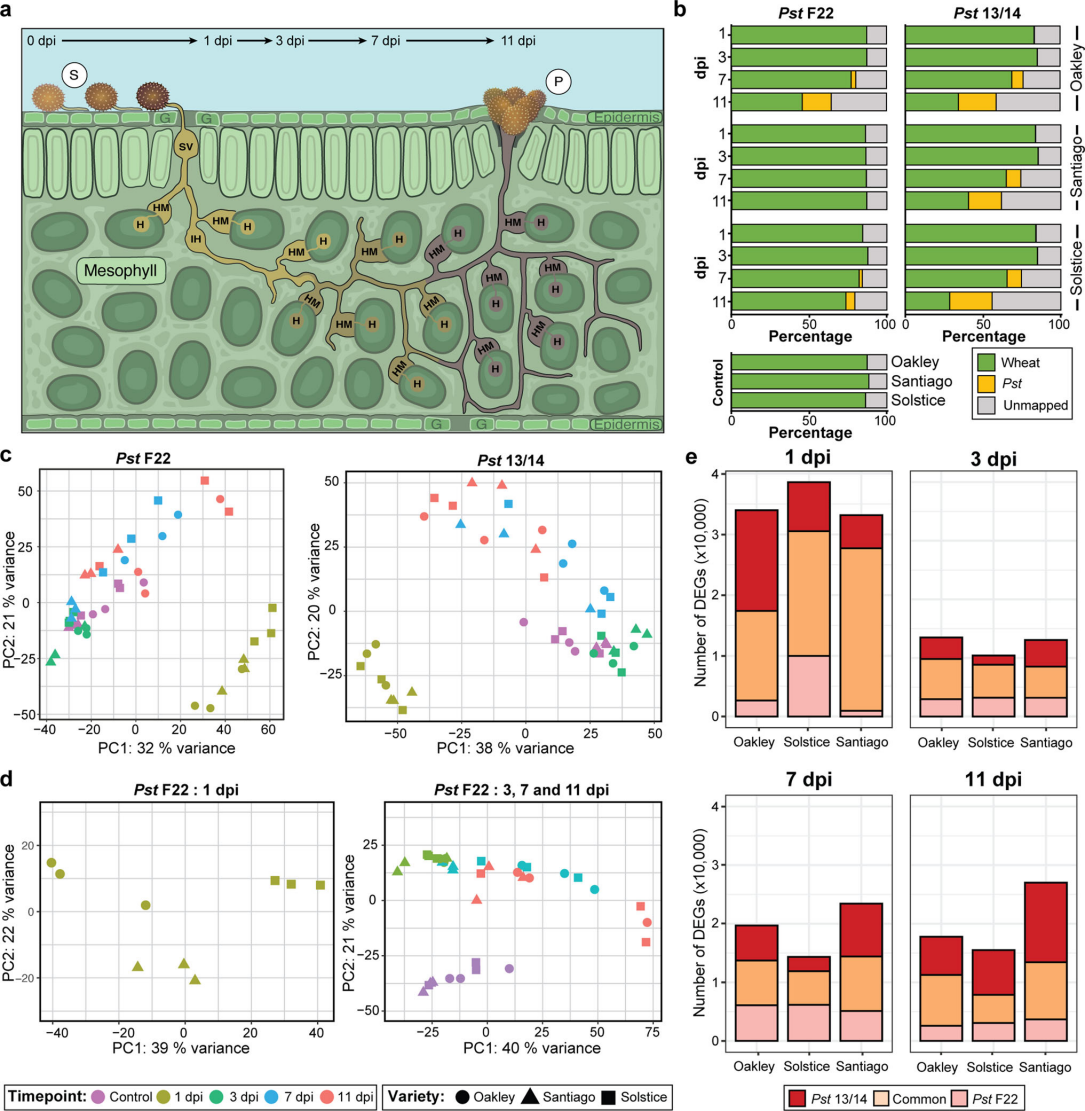
Early stages of *Pst* infection induce massive transcriptional reprogramming of the wheat host, irrespective of its susceptibility level. **a** Diagram of the stages of *Pst* development during plant infection. The time points selected for RNA-seq analyses (1, 3, 7 and 11 days post-inoculation [dpi]) are highlighted. S uredinospore, SV substomatal vesicle, IH invasive hyphae, HM haustorial mother cell, H haustorium, P pustule, G guard cell. Inspired by a schematic illustration from^61^. **b** Percentage of reads mapping to the wheat or *Pst* reference genomes across wheat varieties and pathogen isolates. Following quality filtering, RNA-seq reads were mapped to the *Pst* reference genome (isolate *Pst*-104E^18^) and wheat reference genome Refseq v1.1^17^. Values represent an average of three independent biological replicates (independent infected plants) for each *Pst*–variety pair. **c** Principal component analysis (PCA) of wheat gene expression profiles shows that samples from all *Pst*–variety pairs cluster into two well-defined groups: 1) 1 dpi; and 2) all remaining time points. **d** Independent PCA on 1 dpi samples only (left) or remaining time points (right) illustrating the clustering of 1 dpi samples by host variety, for infection by *Pst* isolate F22. **e** Differentially expressed genes (DEGs) are more numerous at 1 dpi, with samples infected with *Pst* isolate 13/14 showing more isolate-specific DEGs than those infected with *Pst* isolate F22. The number of DEGs was defined at each time point by comparing normalised transcript abundance for each *Pst*-wheat interaction against the corresponding mock-inoculated control using a negative binomial regression (Wald test) in DESeq2. Genes were considered differentially expressed when *q*-value < 0.05.

We selected three bread wheat varieties (Oakley, Solstice and Santiago) previously demonstrated to display different susceptibility levels to our two selected *Pst* isolates (F22 and 13/14)^14^. We quantified visible phenotypes of pathogen infection and infection types (ITs) at 12 days post-inoculation (dpi) following the 0–4 scale^16^ (Supplementary Table S1). Oakley was fully susceptible to both *Pst* isolates, while Solstice was moderately susceptible to *Pst* isolate F22 and almost fully susceptible to *Pst* isolate 13/14; Santiago was resistant to *Pst* isolate F22 and showed moderate resistance to *Pst* isolate 13/14. These results confirmed the range of susceptibility/resistance exhibited by the selected wheat varieties for this study. We infected each of the three wheat varieties with each of the two *Pst* isolates individually (Fig. 1a) and collected samples at 1, 3, 7 and 11 dpi for RNA-seq analysis, alongside mock-inoculated samples from each variety collected at 12 h post-inoculation (hpi). Following quality filtering, we aligned clean reads from each of the 81 generated samples to the wheat reference genome (Refseq v1.1)^17^ and *Pst* reference genome (isolate *Pst*-104E^18^).

We detected similar proportions of reads mapping to the wheat and *Pst* reference genomes across samples collected at 1 and 3 dpi (average of 85.5 ± 1.5% for wheat and < 1% for *Pst*, Fig. 1b). By 7 dpi, the percentage of reads mapping to the wheat and *Pst* genomes varied and reflected the degree of susceptibility between the respective variety–pathogen pairs. We observed the largest differences between varieties at 11 dpi upon infection with *Pst* isolate F22. Indeed, while we obtained an average of 45.7 ± 24.0% reads mapping to wheat and 18.7 ± 11.5% to *Pst* for the most susceptible interaction (*Pst* isolate F22–Oakley), the fraction of reads mapping to *Pst* decreased with higher host resistance. The moderately susceptible interaction (*Pst* isolate F22–Solstice) returned 73.6 ± 20% of reads mapping to wheat and 5.76 ± 8.02% to *Pst*, compared to 87.0 ± 0.92% of reads mapping to the wheat genome and 0.05 ± 0.02% to *Pst* in the context of the most resistant interaction (*Pst* isolate F22–Santiago). Notably, the percentages of reads mapping to the wheat genome were comparable for the Santiago–*Pst* isolate F22 pair between early and later time points, as well as with mock-inoculated samples (87.3 ± 1.8%), in agreement with the high resistance of the host to the pathogen (Fig. 1b). By contrast, infection of all three varieties with *Pst* isolate 13/14 resulted in similar percentages of reads mapping to each reference genome (host and pathogen) at 7 and 11 dpi, although samples collected from the highly susceptible variety Solstice showed the largest percentage of reads mapping to *Pst* at 11 dpi relative to the other two varieties (Fig. 1b). This analysis illustrates that the percentage of reads mapping to the wheat and *Pst* genomes at later time points reflect the degree of susceptibility of each *Pst*–variety interaction.

### Wheat gene expression profiles are most notably altered early in the *Pst* infection process

To assess the host response to *Pst* infection under different levels of susceptibility, we determined wheat transcript abundances at each time point for each *Pst*–variety interaction. We normalised our data to account for library size and samples with low read counts before conducting a principal component analysis (PCA). We generated scatterplots of the first two principal components for each *Pst* isolate, which identified two well-defined groups across all *Pst*-infected samples: (1) samples collected at 1 dpi and (2) samples collected at all remaining time points (Fig. 1c). As samples collected at 1 dpi clustered separately from all others and might obscure later transcriptome patterns, we repeated the PCA by separating the 1 dpi samples from the others (Fig. 1d and Supplementary Fig. S1). The scatterplot of the first two principal components for all 1 dpi samples demonstrated a clear separation by *Pst* isolate and wheat variety. We also noticed that separation between wheat varieties tends to follow their genetic relatedness, with Santiago grouping closely with its parent variety Oakley, whereas the unrelated Solstice variety clustered separately (Fig. 1d and Supplementary Fig. S1). Analyses of the remaining time points showed a similar distribution for both *Pst* isolates, with mock-inoculated control samples clustering together and away from the remaining time points (3, 7 and 11 dpi). These results suggest that host transcript abundance is largely similar at 3 dpi onward irrespective of the *Pst* isolate or the level of susceptibility of the wheat variety used for infection.

We identified differentially expressed genes (DEGs) at the different time points by comparing normalised transcript abundance for each *Pst*–variety interaction against their respective mock-inoculated controls. Overall, we observed substantial overlap between DEGs from different *Pst*–variety pairs, ranging from 68.7 ± 13.0% (standard deviation) to 59.5 ± 14.2% shared between *Pst* F22- and *Pst* 13/14-infected samples. In agreement with the PCA, we detected far more DEGs at 1 dpi (*q*-value < 0.05), with an average number of 27,973 ± 5453 DEGs across all *Pst*–variety interactions (Fig. 1e), compared to 9125 ± 1193 at 3 dpi, 13,357 ± 3305 at 7 dpi, and 13,928 ± 5222 at 11 dpi. Looking at *Pst* isolate-specific transcriptional responses, we determined that all wheat varieties exhibit more DEGs specific for *Pst* 13/14 infection than with *Pst* F22 (Fig. 1e and Supplementary Data 1 and 2). This pattern was particularly evident at 1 dpi, with 30,733 ± 1886 DEGs across the three varieties infected with *Pst* 13/14, of which 10,035 ± 5825 were unique to *Pst* 13/14. Conversely, across the three varieties infected with *Pst* F22 a total of 25,213 ± 6923 DEGs were identified, of which 4516 (range 933–9987) were specific to *Pst* F22 at 1 dpi. Notably, 96.6% of all DEGs at 1 dpi in Santiago plants infected with *Pst* F22 were also differentially expressed in Santiago infected with *Pst* 13/14, despite the difference in susceptibility (resistance for *Pst* F22, moderately resistant for *Pst* 13/14).

### Gene expression profiles reflect the variety and *Pst* isolate early during infection

**Fig. 2:**
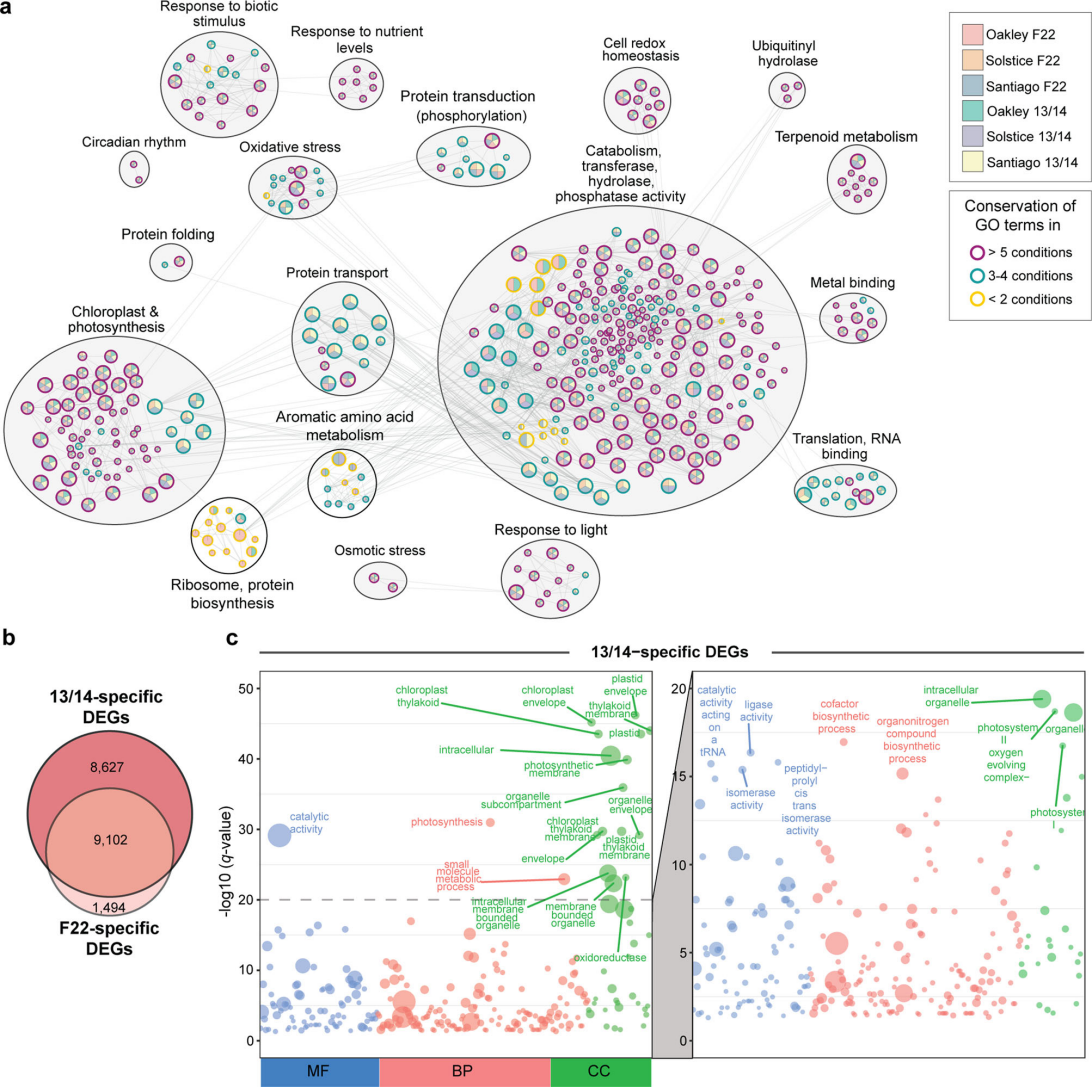
A larger number of biological processes were enriched at the earliest time point following infection with *Pst* isolate 13/14. **a** Functional enrichment network for each *Pst*–variety pair identified in samples taken at 1 dpi. Gene ontology (GO) terms were assigned to all DEGs where possible and those identified as significantly enriched (*q*-value < 0.0005) in at least one *Pst*-varietal pair are represented by a node, with node sizes proportional to the number of genes annotated with the GO term. Edges indicate overlapping member genes and conservation of GO term enrichment is highlighted by node border colour. Highly similar gene sets formed clusters, which were annotated and labelled with appropriate summarising terms. **b** Venn diagram illustrating the extent of overlap between the number of DEGs conserved for the three wheat varieties at 1 dpi upon inoculation with *Pst* isolate 13/14 or *Pst* isolate F22. **c** Functional GO term enrichment analysis results for the 8627 *Pst* 13/14-specific DEGs. GO terms were annotated when –Log(*q*-value) > 20 (first panel) or –Log(*q*-value) > 15 (second panel). Circle size represents the number of genes annotated within the particular enriched function; circle colour represents the GO term classification: molecular function (MF, blue), biological process (BP, pink) and cellular component (CC, green).

To identify biological processes associated with variety-specific expression profiles in response to *Pst* infection, we generated functional enrichment networks for each *Pst*–variety pair (Fig. 2a and Supplementary Figs. S2 and S3). Accordingly, we assigned gene ontology (GO) terms to all DEGs where possible and identified those significantly enriched in each condition (*q*-value > 0.0005). We detected enrichment for second-level GO terms across all conditions and time points that reflected general responses to *Pst* infection and included GO:0009536 (plastid), GO:0009507 (chloroplast) and GO:0003824 (catalytic activity) (Fig. 2a and Figs. S2 and S3). Focusing on DEGs at 1 dpi, all *Pst*–variety pairs showed enrichment in functions related to response to biotic stimulus, chloroplast and photosynthesis, metal binding (iron–sulfur cluster binding), cell redox homoeostasis and cell metabolism, including transferase activity, hydrolase activity and phosphatase activity (Fig. 2a). Looking across all wheat varieties, we identified 1494 DEGs specifically in response to infection with *Pst* F22 and another 8627 DEGs specific to inoculation with *Pst* 13/14 (Fig. 2b). Functional annotation of each set of DEGs highlighted functions related to protein transport and protein localisation for those specific to *Pst* F22 infection (Fig. S4), while those specific to *Pst* 13/14 infection were related to part of the chloroplast, the chloroplast membrane and photosystems (Fig. 2c).

**Fig. 3:**
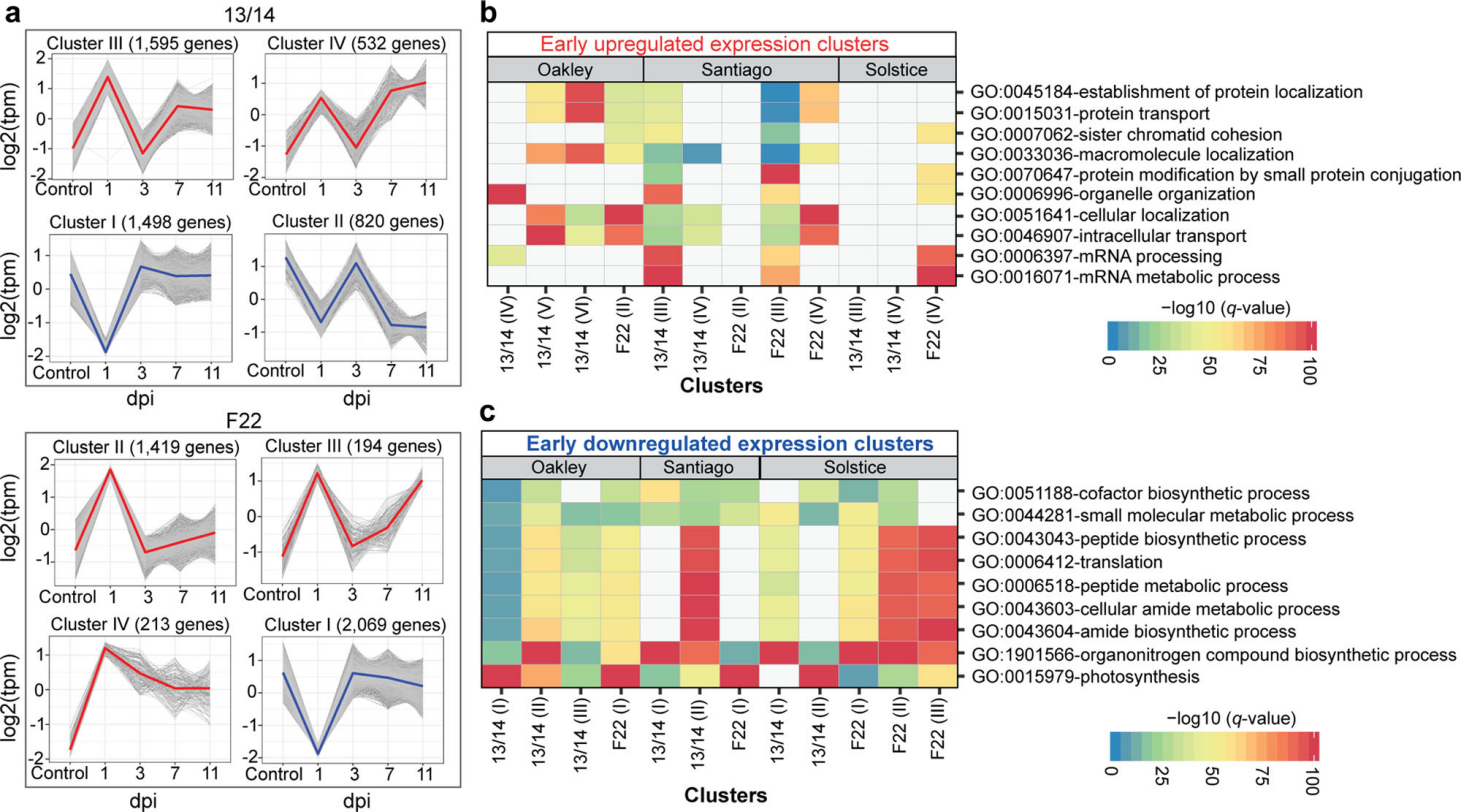
Co-expression clusters for the wheat genes upregulated or downregulated early during *Pst* infection. **a** Example of co-expression clusters classified as containing ‘early upregulated’ (red) or ‘early downregulated’ (blue) genes following infection of Santiago with *Pst* isolates 13/14 and F22. Co-expression clusters were generated using the 8627 *Pst* 13/14-specific DEGs. The coloured line represents the average normalised expression of all genes in a given co-expression cluster. **b**, **c** GO terms for functionally enriched biological processes across the co-expression clusters from 8627 *Pst* 13/14-specific DEGs, assessed for each *Pst*–variety pair, and classified as ‘early upregulated’ (**b**) or ‘early downregulated’ (**c**) genes. Significant –Log(*q*-value) values are represented using a 0–100 scale and GO terms with –Log(*q*-value) > 5 are shown.

We hypothesised that the greater number of DEGs shared across wheat varieties infected with *Pst* 13/14 reflects either the more homogeneous susceptible phenotypes or the stronger transcriptional reprogramming induced by this isolate. To explore this question in more detail, we built co-expression clusters for each *Pst–*variety pair by using the 8,627 DEGs identified at 1 dpi (Figs. S5–S10). We classified the clusters into two classes based on expression profiles: (1) ‘early upregulated’ clusters whose constituent genes were highly expressed at 1 dpi but returned to mock-inoculated levels by 3 dpi and (2) ‘early downregulated’ clusters whose genes were expressed at lower levels than the controls at 1 dpi but returned to mock-inoculated levels by 3 dpi. For example, during the fully susceptible interaction between Santiago and *Pst* 13/14, we classified 2127 genes across two co-expression clusters as ‘early upregulated’ and 2318 genes from two co-expression clusters as ‘early downregulated’. Using the same method in the context of the resistant interaction between Santiago and *Pst* F22, we identified 1826 genes across three co-expression clusters as ‘early upregulated’ and 2069 genes from one co-expression cluster as ‘early downregulated’ (Fig. 3a).

GO term enrichment analysis indicated that ‘early upregulated’ DEGs are associated with a diverse array of cellular processes (Figs. 3b and S11). All co-expression clusters for each of the three varieties infected with *Pst* 13/14 contained genes mainly involved in the myosin complex and peroxisomes. The resistant interaction (*Pst* isolate F22–Santiago) was the only one associated with the NatA acetyltransferase complex, which also contained genes involved in protein deubiquitination. In terms of biological processes, early upregulated genes in the context of resistant and moderately susceptible interactions included mRNA metabolism and protein modification by small protein conjugation or removal. Susceptible interactions comprised genes involved in organelle organisation, protein transport, RNA processing, protein modification and pyridine nucleotide salvage. By contrast, we observed shared functions across all conditions for genes classified as ‘early downregulated’ (Figs. 3c and S12). In terms of cellular components, these co-expression clusters included genes annotated as part of the chloroplast. In agreement with this observation, photosynthesis was the main biological process enriched in all clusters, with other enriched processes such as organonitrogen compound biosynthesis, peptide metabolism and translation. Notably, the specific early downregulated genes associated with the chloroplast and involved in photosynthesis differed between each *Pst*–variety pair.

### Global gene expression analysis reveals modulation of host defence-related genes

**Fig. 4:**
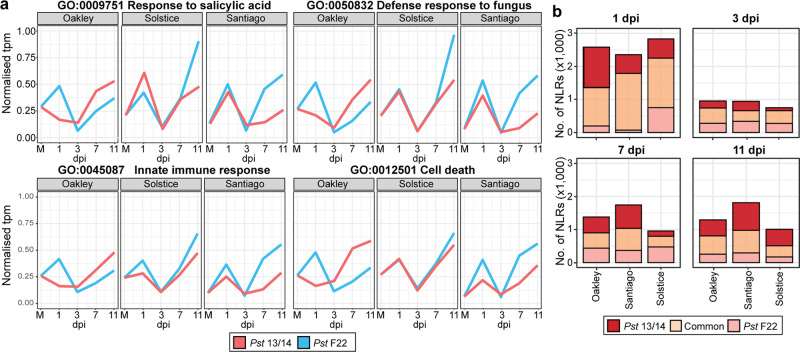
Genes involved in host defence-related responses show different expression patterns across different *Pst*–variety pairs and reflect host susceptibility. **a** Median expression of normalised transcripts per million (tpm) values obtained for genes annotated as being involved in response to salicylic acid (GO:0009751), defence response to fungus (GO:0050832), innate immune response (GO:0045087) and cell death (GO:0012501). The peak in gene expression at later stages of infection (7–11 dpi) is more pronounced in resistant interactions (*Pst* isolate F22–Santiago) when compared to its susceptible counterpart (*Pst* isolate 13/14–Santiago). **b** The number of DEGs encoding proteins with typical NLR domains is greatest at 1 dpi, with the most DEGs at this time point identified in samples from Oakley infected with *Pst* 13/14 (most susceptible interaction). Typical NLR domains were defined as IPR001611:Leu-rich_rpt, IPR032675:LRR_dom_sf, IPR002182:NB-ARC, IPR027417:P-loop_NTPase. Genes were considered differentially expressed compared to the control when *q*-value < 0.05.

Among the DEGs at 1 dpi, we observed an enrichment for functions associated with defence-related responses. We selected genes participating in programmed cell death (48 genes), response to salicylic acid (SA; 59 genes), the innate immune response (179 genes), defence response to fungi (151 genes) and those predicted to encode nucleotide-binding site leucine-rich repeat (NLR)-type R proteins (9078 genes) for further analysis. We normalised their expression and determined the median value (Fig. 4a, b). Most varieties exhibited a consistent upregulation of transcript levels across all categories at 1 dpi, followed by a drop in expression at 3 dpi and a later increase at 7 and 11 dpi. Importantly, the expression of genes belonging to all four defence-related response processes reaches a higher peak at later stages of infection (7–11 dpi) in the resistant interaction (*Pst* isolate F22–Santiago) relative to its susceptible counterpart (*Pst* isolate 13/14–Santiago) (Fig. 4a). Turning to genes annotated as encoding potential NLRs, we detected most DEGs from this class at 1 dpi. At this time point, we identified the greatest numbers of NLR DEGs for Oakley infected with *Pst* 13/14 (most susceptible interaction), followed by Solstice infected with *Pst* 13/14 (fully susceptible) and *Pst* F22 (moderate susceptibility). The lowest numbers of NLR DEGs were for Santiago infected with *Pst* F22 (resistant interaction) and Oakley infected with *Pst* F22 (fully susceptible) (Fig. 4b). However, we noted that at 1 dpi in Oakley infected with *Pst* F22, many genes involved in defence-related responses lacked the expression peak seen in other *Pst*–variety pairs, likely due to the peak occurring outside of the sampling timepoint in this case. Overall, our results suggest that the outcome of the host–pathogen interaction may be decided early during initial fungal colonisation.

### The expression of nuclear genes encoding chloroplast proteins (NGCPs) is temporally coordinated in response to *Pst* infection

**Fig. 5:**
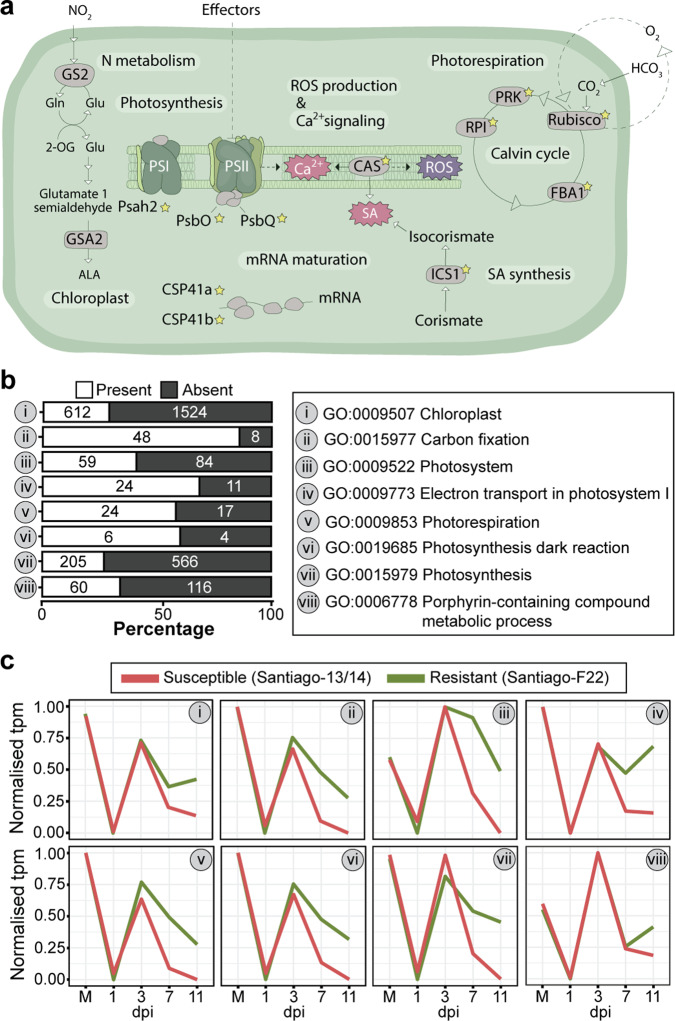
Nuclear genes encoding chloroplast-localised proteins (NGCPs) are differentially expressed early during *Pst* infection and have temporarily coordinated expression profiles. **a** Schematic illustration of the chloroplast. The genes encoding the proteins marked with a star were identified as differentially expressed at 1 dpi across wheat varieties upon infection with *Pst* isolate 13/14. **b** Many genes are annotated with chloroplast-related functions among the 8627 *Pst* 13/14-specific DEGs, as 1038 DEGs belong to eight second-level GO terms with chloroplast-related functions. **c** Chloroplast-related DEGs show a conserved, temporally regulated expression profile during *Pst* infection. Normalised transcripts per million (tpm) values were used to determine the median expression levels for genes assigned to each of the eight chloroplast-related GO terms.

Among the early downregulated genes, we noticed the presence of many genes encoding proteins with GO terms associated with the chloroplast (Fig. 3c). We identified components of photosystem I (Psah2) and II (PsbQ proteins and PsbO2), enzymes from the Calvin–Benson–Bassham cycle (pyruvate kinase [PRK], Ribose-5-phosphate isomerase [RPI], Rubisco, Fructose-bisphosphate aldolase [FBA1]), chloroplast calcium signalling components (CAS), proteins involved in chloroplast RNA metabolism (CSP41a and CSP41b) and isochorismate synthase 1 (ICS1) that synthesises SA in the chloroplasts from chorismic acid (Fig. 5a). In each case, their gene expression was downregulated at 1 dpi, followed by a sharp peak in expression at 3 dpi and a second rapid decline by 7 dpi. The most resistant interaction (*Pst* isolate F22–Santiago) was the notable sole exception across *Pst*–variety pairs, as the expression of many of these genes, failed to decline or declined to a lesser extent after 3 dpi than with more susceptible interactions (Supplementary Fig. S13).

We explored the expression patterns of these nuclear genes encoding chloroplast-localised proteins (NGCPs) during a susceptible *Pst*-wheat interaction by re-examining the enriched GO terms among the 8627 *Pst* 13/14-specific DEGs. We obtained 1038 DEGs that belong to eight second-level GO terms with chloroplast-related functions. For each of the eight categories, we determined the genome-wide number of genes associated with each GO term, which illustrated the high proportion of chloroplast-related genes among the 8627 DEGs (26.6–85.7% for each GO term) (Fig. 5b and Supplementary Data 3). In addition, all chloroplast-related genes followed the same pattern of expression observed above, with a sharp increase in expression at 3 dpi, followed by a rapid decline by 7 dpi, except in the highly resistant interaction (*Pst* isolate F22–Santiago; Fig. 5c). This conserved gene expression profile likely reflects a well-coordinated transcriptional modulation of genes encoding chloroplast-targeted proteins upon pathogen recognition.

### *TaCSP41a* expression is altered early during *Pst* infection and likely encodes a chloroplast protein

**Fig. 6:**
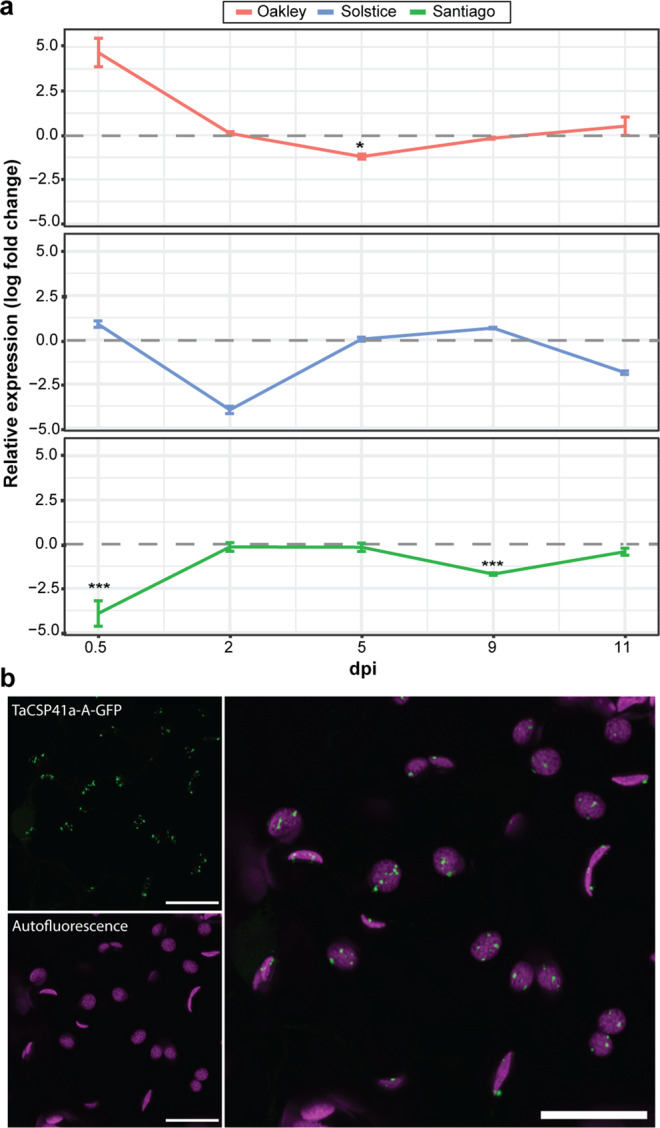
*TaCSP41a* encodes a putative chloroplast-localised protein and its expression early during *Pst* infection is linked to host susceptibility. **a** *TaCSP41a* expression during a controlled infection time course of the wheat varieties Oakley, Solstice and Santiago with *Pst* isolate F22. Relative *TaCSP41a* expression was measured by RT-qPCR from all three homoeologous copies simultaneously and compared to mock-inoculated control plants, with the *UBC4* gene used as a reference^53^. Two independent leaves from the same plant were pooled and three independent plants were analysed for *TaCSP41a* expression at each time point. Asterisks denote statistically significant differences (***p < 0.005, **p < 0.01, *p < 0.05; 2-tailed *t*-test). **b** TaCSP41a-A co-localises with chlorophyll autofluorescence. *TaCSP41a-A-GFP* was transiently expressed in *N. benthamiana* and images were captured after 2 days. Images are representative of >10 images captured, all displaying co-localisation of TaCSP41a-A-GFP and chlorophyll autofluorescence. Left, individual TaCSP41a-A-GFP (top) and chlorophyll autofluorescence (bottom) patterns; right, merged image of TaCSP41a-A-GFP and chlorophyll autofluorescence illustrating co-localisation. Scale bars, 10 μm.

We selected the putative chloroplast-localised stem-loop RNA binding protein TaCSP41a among NGCPs for detailed analyses. TaCSP41a was selected due to the availability of tetraploid Kronos TILLING mutants and as CSP41 abundance has previously been linked to abiotic stress in *Arabidopsis* and tomato (*Solanum lycopersicum*)^19,20^. To investigate the expression pattern of *CSP41* in more detail in response to biotic stress, we performed an RT-qPCR analysis of *TaCSP41a* transcript levels at 12 hpi, 2, 5, 9 and 11 dpi following infection of the wheat varieties Oakley, Santiago and Solstice with *Pst* F22. We designed primers to amplify all three *TaCSP41a* homoeologues simultaneously and compared expression levels between infected and mock-inoculated plants (Fig. 6a). *TaCSP41a* was substantially more highly expressed at 12 hpi in the highly susceptible variety Oakley and expressed significantly lower levels in the highly resistant variety Santiago upon infection (Fig. 6a). In all susceptible interactions, *TaCSP41a* was initially more highly expressed before decreasing substantially, reaching its lowest levels by 5 dpi for infected Oakley and 2 dpi for infected Solstice. These observations confirmed a link between *TaCSP41a* expression early during infection and the extent of susceptibility to *Pst* infection as shown in the RNA-seq analyses.

To test the subcellular location of TaCSP41a, we scanned the predicted protein sequence of the three homoeologues TaCSP41a-A, TaCSP41a-B, TaCSP41a-D for potential targeting signals. We detected a chloroplast targeting peptide with a high probability (>99%) in all three homoeologues (Supplementary Table S2). Encouraged by this result, we generated a fusion construct by cloning the *TaCSP41a-A* coding sequence in-frame and upstream of that of the green fluorescent protein (GFP) and transiently infiltrated the resulting *TaCSP41a-A-GFP* construct in *Nicotiana benthamiana* leaves. We observed GFP fluorescence in foci that co-localise with chlorophyll autofluorescence, as determined by confocal microscopy, supporting the notion that TaCSP41a is a chloroplast-resident protein (Fig. 6b).

### Disruption of *TaCSP41a* restricts *Pst* infection

**Fig. 7:**
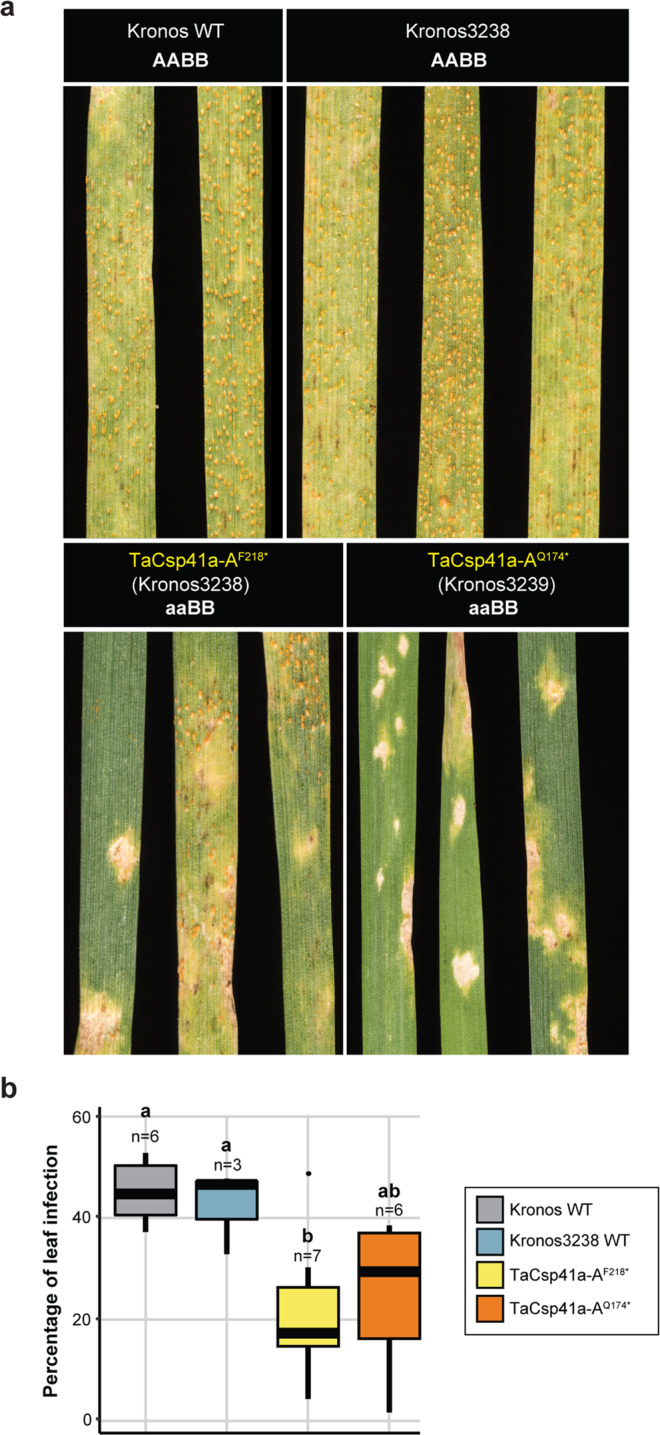
*TaCSP41a* disruption mutants are more resistant to *Pst* infection. **a** *TaCSP41a-A*^F218*^ and *TaCSP41a-A*^Q174*^ disruption mutants are more resistant to infection by *Pst* isolate 13/14 compared to the Kronos wild type (WT) or the Kronos ethyl methanesulfonate (EMS) mutant Kronos3238 carrying a WT allele at *TaCSP41a*. Images were captured at 20 dpi. **b** Lower rates of leaf infection in the *TaCSP41a-A* disruption mutants at 20 dpi, represented as box and whiskers plots. Lowercase letters denote statistically significant differences by Duncan’s multi-range test (*p* < 0.05). Horizontal bars, median values; boxes, upper (Q3) and lower (Q1) quartiles; whiskers, 1.5 × the inter-quartile range.

To assess the contribution of *TaCSP41a* to *Pst-*induced disease progression, we looked for tetraploid Kronos TILLING mutants^21^. We identified two mutant lines (Kronos3238 and Kronos3239) introducing early stop codons in the *TaCSP41a-A* sequence at amino acids 218 and 174 (Supplementary Fig. S14). We obtained homozygous TILLING mutant lines by self-pollination. We infected F_2_ homozygous progeny (*TaCSP41a-A*^F218*^ and *TaCSP41a-A*^Q174*^) with *Pst* 13/14 and compared their disease phenotypes to the wild type (WT, *cv*. *Kronos*) and a Kronos3238 sibling carrying the wild-type allele at *TaCSP41a-A* (Fig. 7a). Both mutant lines displayed limited sporulation and higher *Pst* resistance at 20 dpi, with a substantial reduction in the extent of leaf area infected by *Pst*, compared to both the Kronos WT and the wild-type Kronos3238 sibling (Fig. 7b). Leaves of the *TaCSP41a-A*^F218*^ and *TaCSP41a-A*^Q174*^ mutant lines remained largely green outside of a few necrotic spots consistent with localised programmed cell death. By contrast, both WT lines were uniformly chlorotic, with low or no necrotic lesions (Fig. 7a). The *TaCSP41a-A*^Q174*^ mutant line displayed a stronger phenotype, with no chlorosis and only small necrotic regions in all plants tested. Together, these results demonstrate that disrupting TaCSP41a-A function promotes tolerance to *Pst* 13/14, indicating a role for TaCSP41a in supporting *Pst* disease progression.

## Discussion

Exploring host transcriptional reprogramming can provide insight into plant defence responses during infection and the host molecular mechanisms targeted by pathogens to support disease progression. To identify such host genes in wheat, we generated RNA-seq datasets spanning the entire infection cycle by two *Pst* isolates (13/14 and F22) with contrasting virulence on three selected commercial wheat varieties (Oakley, Solstice and Santiago) with different levels of susceptibility^14^. Our approach is more expansive than previous efforts, which were geared toward binary comparisons using single historical *Pst* isolates, characterising the wheat host response during race-specific compatible and incompatible interactions, and generally focusing on a limited number of time points typically early during infection^22–25^. During early time points (1 and 3 dpi), we established that the number of reads mapping to the pathogen genome is comparable across all *Pst*–variety combinations, independently of susceptibility. These early time points were also characterised by fewer than 1% of all reads mapping to the fungal genome, reflecting the early stage of infection. This low apparent titre of the pathogen is consistent with previous observations of *Pst*-wheat infections^23^ and in response to other biotrophic fungal pathogens such as *Ustilago maydis* infecting maize^26^. Notably, although we detected very few reads mapping to the *Pst* reference genome at the earliest time point (1 dpi), we nevertheless identified the greatest number of host DEGs for each *Pst*–variety combination, including for DEGs encoding proteins with typical NLR domains, which were more numerous at 1 dpi than at any other time point. *Pst* infection, therefore, triggers the greatest alteration in the host gene expression programme at the initial stages of fungal penetration.

In this study, the cross-comparison across wheat varieties with differing susceptibility to *Pst* facilitated the dissection of the correlations between the expression of host defence response genes and disease outcomes. Host defence response genes were generally upregulated very early during infection (1 dpi) in a largely synchronised manner across varieties, with the exception of the highly resistant interaction between Santiago and *Pst*. This result is similar to observations made across different potato (*Solanum tuberosum*) varieties in response to *Phytophthora infestans* infection, where the expression of defence-related genes was induced across varieties, but their temporal regulation differed between susceptible and resistant lines^27^. Previous analyses of defence-related genes (NLR-encoding genes) in the context of the *Pst*-wheat interaction had similarly demonstrated that gene expression is dramatically suppressed by 3 dpi in susceptible varieties, but not in a resistant interaction^23^. We will note here that we did not observe a high and early peak in expression for many defence response genes for the fully susceptible interaction between *Pst* F22 and Oakley, perhaps because the genes reached their highest expression levels outside of the time points analysed here.

We also determined that NGCPs were highly enriched in all *Pst–*variety pairs and displayed a largely synchronous temporal regulation in their expression. Genes participating in photosynthesis and terpenoid-quinone biosynthesis have been reported to be induced during early pathogen recognition in wheat upon infection with both *Pst* and *Bgt*^24^. In addition, transient silencing of one NGCP, *TaClpS1*, was shown previously to reduce *Pst* infection in wheat^28^. Furthermore, both bacterial and fungal effectors have been reported to localise to the chloroplast *in planta*^29–33^. This pattern of accumulation highlights the potential for pathogen effectors to target functions inside chloroplasts. For instance, the biosynthesis of SA, a key phytohormone for innate immunity^34^, in plastids can be modulated by pathogen effectors that hydrolyse or modify SA precursors to suppress host immunity^35^, as with the *Ustilago maydis* effector Cmu1^36^. Fungal effectors can also specifically target NGCPs to suppress their function by inhibiting entry into chloroplasts, as with the *Pst* effectors Pst_4 and Pst_5^37^. Pathogen perception has also been reported to trigger a rapid suppression of expression of NGCPs in other pathosystems, leading to the production of reactive oxygen species (ROS) that restrict pathogen growth^38^. Here, we showed that NGCPs are characterised by very low expression levels by 1 dpi across all *Pst* isolate and variety pairs. However, it is difficult to distinguish between an early suppression of photosynthetic functions as a plant response to limit carbon availability to the pathogen or activation by the pathogen to weaken the plant and support colonisation. Pathogen effectors such as HopN1 from the bacterial pathogen *P. syringae* block cell death, ROS production, and callose deposition by physically interacting with PsbQ in thylakoids in *Arabidopsis*, likely to induce its proteolysis^30^. Therefore, the later increase in NGCP expression we observed by 3 dpi, which is consistent with prior reports of stimulation in the photosynthetic rate at 48 h post-Pst infection^39^, may reflect a host plant compensatory mechanism for the loss of photosynthetic components.

We detected a final decline in NGCP expression at 7 dpi that was much more pronounced in the susceptible interaction (*Pst* isolate 13/14–Santiago) compared to the same variety involved in a resistant interaction (*Pst* isolate F22–Santiago). At 7 dpi, we also noted that the expression of genes involved in photosynthesis is induced in all *Pst–*variety pairs, especially in the resistant interaction (*Pst* isolate F22–Santiago). This observation may suggest reactivation of the photosynthetic machinery that remains suppressed in susceptible interactions during the late stages of biotrophic fungal growth. Among the NGCPs identified here, we noted the wheat *Isochorismate Synthase 1* (*TaICS1*) homologue, whose transcript levels increased at 3 dpi, especially in the context of a highly resistant interaction (*Pst* isolate F22–Santiago) (Supplementary Fig. S13). In *Arabidopsis*, pathogen infection raises *AtICS1* expression levels and thus promotes SA-mediated defence in resistant interactions^40^. Overall, our results illustrate that NGCPs expression is highly coordinated during *Pst* infection and highlights chloroplast-related functions as prime targets of *Pst* manipulation.

Although NGCP expression was highly coordinated, it also differed at later infection stages between susceptible and resistant interactions, prompting us to investigate their potential role in supporting pathogen progression. Among NGCPs with a distinct expression profile between the two types of host–pathogen interactions, we selected *TaCSP41a*, encoding a potential chloroplast-localised stem-loop RNA binding protein, for further analysis. Disruption of the A homoeologue of *TaCSP41a* in the tetraploid wheat *cv*. Kronos led to a strong reduction in *Pst* infection, demonstrating that TaCSP41a contributes to supporting *Pst* infection. CSP41 is absolutely conserved across photosynthetic eukaryotes; *Arabidopsis* CSP41a also accumulates and functions in the chloroplast^41^. CSP41 proteins are likely to be metal-dependent ribonucleases that bind RNA and are critical to the proper regulation of chloroplast transcription, translation and RNA degradation^42^. CSP41 protein abundance increases upon exposure to abiotic stress, as shown by proteomics studies in *Arabidopsis* and tomato^19,20^. Alterations in RNA processing and maturation capacity in the chloroplast may affect the relative balance between chloroplast-translated and cytosol-translated proteins driving the key protein complexes and processes residing in the organelle, such as photosynthesis. Notably, a candidate effector from *Pst* was shown to accumulate in processing bodies containing protein complexes involved in mRNA maturation, degradation and storage^32^, further illustrating how RNA processing may act as a target for manipulation by *Pst* to support its proliferation. Our analysis also highlights how disrupting proteins involved in RNA processing, such as TaCSP41a, can significantly raise resistance against *Pst* infection, which offers a new promising target to be further explored in plant breeding to control wheat yellow rust.

## Methods

### *Pst* inoculation of wheat seedlings and sample collection

A total of 60 seeds from each of the bread wheat (*Triticum aestivum*) varieties Oakley, Santiago and Solstice were pre-germinated, sown in cell trays and grown in a controlled environment consisting of long-day conditions (16-h light/8-h dark) with a light intensity of approximately 250 μmol m^−2^ s^−1^ under a 19 °C/14 °C temperature cycle (day/night). Once seedlings reached the two-week stage, each set of 60 plants per variety was infected with approximately 10 mg of fresh *Pst* urediniospores from isolates 13/14 or F22. To facilitate inoculation, *Pst* urediniospores were heat-activated at 40 °C for 5 min and resuspended in Novec 7100 (Sigma-Aldrich, USA) (1 mg/mL). For control experiments, a mock spray inoculation with Novec 7100 was performed. Immediately following inoculation or mock treatment, seedlings were kept in the dark at 10 °C, with high relative humidity for 24 h before being returned to controlled growth conditions. Infection types (ITs) were assessed 20 days post-inoculation (dpi) in up to 10 plants per variety following the 0–4 scale^16^. Three biological replicates (three different plants) were collected per time point (1, 3, 7 and 11 dpi) for each wheat variety–*Pst* isolate pair. Plant samples were snap-frozen in liquid nitrogen and stored at –80 °C.

### RNA extraction, sequencing and initial filtering of RNA-seq data

Infected leaf material (80–100 mg) was manually disrupted and total RNA was extracted using the Qiagen Plant RNeasy Mini kit (Qiagen, UK) according to the manufacturer’s instructions. Genomic DNA contamination was removed using a TURBO DNA-free Kit (Ambion, UK). The quantity and quality of the RNA were assessed using an Agilent 2100 Bioanalyzer (Agilent Technologies, UK). RNA samples were sent to GENEWIZ (UK) for cDNA library preparation using an Illumina TruSeq RNA Sample Preparation Kit (Illumina, US) and sequencing on an Illumina HiSeq 2500 instrument. The resulting 150-bp paired-end reads were quality-trimmed and filtered using the package fastp v.0.12.3^43^ with default parameters (Phred quality ≥ 15; percent unqualified bases = 40; low complexity filter = 30%; adaptor trimmed by overlap analysis).

### Transcript quantification and differential gene expression analysis

Transcript abundance was assessed for each of the 81 *Pst*-infected and mock-inoculated Oakley, Solstice and Santiago samples following pseudoalignment to the wheat reference transcriptome (RefSeq v1.1 from the cultivar Chinese Spring^17^) using kallisto version 0.43.0^44^. Raw reads from each sample were independently filtered using BBDUK from BBTools version 37.68^45^ and also aligned to the *Pst* reference genome (isolate *Pst*-104E^18^) using STAR version 2.5.a^46^. Transcript per million (tpm) counts were imported into R using tximport version 1.8.0. Principal component analysis (PCA) and differential gene expression analysis were performed using normalised counts in the R package DESeq2 version v.1.16.1^47^. Pairwise comparisons were performed for each *Pst*-infected sample against its appropriate mock-inoculated sample and log_2_ fold-change (LFC) values and Benjamini and Hochberg-adjusted *p*-values were determined. Genes were considered to be differentially expressed when adjusted *p*-values (*q*-value) < 0.05.

### Gene ontology (GO) term enrichment analysis and co-expression clustering

GO term enrichment analysis was performed as described previously^48^. In brief, the g:GOSt tool in g:Profiler^49^ was used with the g:SCS algorithm and a *p*-value cutoff for overrepresentation of <0.05. For enrichment analysis at each time point for individual cultivar–*Pst* isolate pairs, visualisation of GO term networks was performed with the Cytoscape (v3.7) Enrichment Map plug-in (v3.2.1^50^) using a node false discovery rate (FDR) *q*-value cutoff of <0.0005. Clusters of gene sets were annotated according to their GO term annotation using Cytoscape Autoannotate (v1.3.2) and Word Cloud (v3.1) plug-ins. Gene lists for specific GO terms or genes carrying specific domains were obtained using EnsemblPlants BioMart^51^ with wheat gene annotation (RefSeq v1.1^17^). A total of 8627 differentially expressed genes (DEGs) were identified at 1 dpi that were specific to infection with *Pst* isolate 13/14, whose log_2_-normalised tpm values were used to build co-expression clusters using Clust^52^ with a tightness level of 0.5 (−t 0.5).

### Reverse transcription-quantitative PCR (RT-qPCR)

Total RNA was extracted from leaf samples as part of the infection time course performed on the wheat varieties Oakley, Santiago and Solstice inoculated with *Pst* isolate F22. Two independent leaves from the same plant were pooled and three biological replicates (different plants) were collected per time point at 12 h post-inoculation (hpi) and 2, 5, 9 and 11 dpi. Total RNA was extracted as described above and first-strand cDNA synthesised using SuperScript™ II Reverse Transcriptase (Invitrogen, USA), using random hexamers and Oligo(dT) primers according to the manufacturer’s instructions. qPCR was performed on a LightCycler 480 (Roche, Switzerland) using LightCycler 480 SYBR Green I Master Mix (Roche, Switzerland) following the manufacturer’s instructions with each primer at a final concentration of 0.25 µM. Primer sequences are provided in Supplementary Table S3. *TaCSP41a* expression was compared between *Pst*-infected and mock-inoculated plants for each time point and variety, with the *UBC4* gene used as a reference^53^.

### Selection of TILLING wheat lines and KASP genotyping

Two independent tetraploid wheat TILLING (targeting in local lesions in genomes) lines (*cv*. Kronos)^21,54^ with mutations in *TaCSP41a-A* were selected. The TILLING lines Kronos3239 and Kronos3238 each carry a C-to-T transition but at different positions, leading to premature stop codons at amino acid 174 or 218, respectively. Leaf fragments from each mutant line were utilised for DNA extraction^55^, alongside the Kronos wild type. Genotypic analysis was carried out using Kompetitive allele-specific PCR (KASP, LGC Genomics, UK) as described previously^56^ using subgenome-specific primer sequences (Supplementary Table S4). Heterozygous single mutant lines were self-pollinated to obtain homozygous mutant lines. The F_2_ progeny (*TaCSP41a-A*^F218*^ and *TaCSP41a-A*^Q174*^) were screened for susceptibility to *Pst* isolate 13/14 using spray inoculation as described above. Disease symptoms were evaluated 20 days after *Pst* infection and the percentage of infection in independent leaves was measured using K-PIE^57^.

### Annotation of *TaCSP41a* and subcellular localisation of TaCSP41a-A-GFP using confocal microscopy

The predicted subcellular localisation of TaCSP41a encoded by the three homoeologues (TraesCS6A02G025700, TraesCS6B02G036400 and TraesCS6D02G029300) was assessed using TargetP^58^ using default parameters (Supplementary Table S2). For subcellular localisation, the C-terminal GFP fusion construct (TaCSP41a-GFP) was generated in a two-step process as follows: the coding sequence of *TaCSP41a-A* was PCR amplified from cDNA prepared from the wheat variety Santiago using primer sequences 5TaCSP41a-A (5’-CTCACAGGTCGCAGCTAC-3’) and 3TaCSP41a-A (5’-GTGCTCTGTGTCATCTGTATG-3’). A second PCR reaction was performed using the resulting *TaCSP41a*-A amplicon as a template to add Gateway-compatible CACC overhangs using the primer sequences 5TaCSP41a-AENTR (5’-CACCATGGCCTTCTCCCCGGCCAC-3’) and 3TaCSP41a-AENTR (5’-GGCGGCGACGCCGACC-3’). The final *TaCSP41a*-*A* amplicon was cloned into the entry vector pENTR/D-TOPO (Invitrogen, USA) and the resulting clone was confirmed by Sanger sequencing (GENEWIZ, UK). The insert was mobilised into pK7WGF2 (Karimi et al.^59^) by Gateway LR recombination (Invitrogen, USA). The resulting *TaCSP41a-GFP* construct was transformed into Agrobacterium (*Agrobacterium tumefaciens*) strain GV3101 and transiently infiltrated in *Nicotiana benthamiana* leaves following methods described previously^60^.

For confocal microscopy, specimens were mounted in water on microscope slides and examined using an LD LCI Plan Apo ×25/NA 0.8 multi-immersion objective on a Zeiss LSM880 Airyscan upright microscope with ZEN2.3 SP1 (Black) acquisition software. GFP was imaged with the 488 nm line of a 35 mW Argon laser at 2% power output and a detection bandwidth of 495–550 nm. Chlorophyll autofluorescence was imaged with a 5 mW 633-nm He–Ne laser also at 2% power output and a detection bandwidth of 660–695 nm. Image stacks with a voxel size of 74 × 74 × 571 nm were taken in Airyscan super-resolution (SR) mode on a 32-channel GaAsP PMT Airyscan array with a pixel dwell time of 0.7 µs and a gain of 650 and 730 for GFP and chlorophyll, respectively. Raw data were processed with default settings resulting in a nominal resolution improvement of 1.7× compared to confocal mode with a pinhole of 1 AU.

### Statistics and reproducibility

A negative binomial regression (Wald test) in DESeq2 was used to define the number of DEGs at each time point, by comparing normalised transcript abundance for each *Pst*–wheat interaction against the corresponding mock-inoculated control. Genes were considered differentially expressed when *q*-value < 0.05. A two-tailed Student’s *t*-test was used to evaluate the significance of differences in *TaCSP41a* expression during a controlled infection time course of the wheat varieties Oakley, Solstice and Santiago with *Pst* isolate F22 (*n* = 3). Duncan’s multi-range test was used to compare the level of *Pst* infection (isolate 13/14) for *TaCSP41a-A*^F218*^ and *TaCSP41a-A*^Q174*^ disruption mutants to the Kronos wild type (WT) or the Kronos ethyl methanesulfonate (EMS) mutant Kronos3238 carrying a WT allele at *TaCSP41a* (*n* = 7, 6, 7, 3, respectively).

### Reporting summary

Further information on research design is available in the Nature Research Reporting Summary linked to this article.

## Supplementary information

Supplementary Information

Description of Additional Supplementary Files

Supplementary Data 1

Supplementary Data 2

Supplementary Data 3

Supplementary Data 4

Reporting Summary

## Data Availability

Sequence data that support the findings of this study can be found in the European Nucleotide Archive (ENA) database under the following accession number: PRJEB50522 and additional data is available in the supplementary material of this article (Supplementary Data 4).
