# Supplementary material for: Temporally coordinated expression of nuclear genes encoding chloroplast proteins in wheat promotes Puccinia striiformis f. sp. tritici infection
Source: Commun Biol. 2022 Aug 22;5:853. doi: 10.1038/s42003-022-03780-4 (PMC9395331; doi:10.1038/s42003-022-03780-4)
Supplement: Supplementary file 1 — Supplementary Information [file 42003_2022_3780_MOESM1_ESM.pdf]

COMMUNICATIONS BIOLOGY: Supplementary Information

Temporally coordinated expression of nuclear genes encoding chloroplast proteins in wheat promotes *Puccinia striiformis* f. sp. *tritici* infection

Pilar Corredor-Moreno, Roshani Badgami, Sally Jones, Diane G.O. Saunders

The following Supplementary Information is available for this article:

Fig. S1. Principal component (PC) analysis illustrated that samples tended to group at the earliest time-point (1 dpi) by variety following infection with *Pst* isolate 13/14.

Fig. S2. A number of biological processes were enriched at 3 and 7 days post-inoculation (dpi).

Fig. S3. A number of biological processes were enriched at 11 days post-inoculation (dpi).

Fig. S4. Gene ontology (GO) terms identified as functional enriched among the 1,494 *Pst* F22-specific DEGs included a large array of biological processes.

Fig. S5. Eleven co-expression clusters were identified among the 8,627 *Pst* 13/14-specific DEGs following infection of Oakley with *Pst* isolate F22.

Fig. S6. Eleven co-expression clusters were identified among the 8,627 *Pst* 13/14-specific DEGs following infection of Santiago with *Pst* isolate F22.

Fig. S7. Fourteen co-expression clusters were identified among the 8,627 *Pst* 13/14-specific DEGs following infection of Solstice with *Pst* isolate F22.

Fig. S8. Fourteen co-expression clusters were identified among the 8,627 *Pst* 13/14-specific DEGs following infection of Oakley with *Pst* isolate 13/14.

Fig. S9. Eleven co-expression clusters were identified among the 8,627 *Pst* 13/14-specific DEGs following infection of Santiago with *Pst* isolate 13/14.

Fig. S10. Twelve co-expression clusters were identified among the 8,627 *Pst* 13/14-specific DEGs following infection of Solstice with *Pst* isolate 13/14.

Fig. S11. Cellular processes enriched for genes belonging to co-expression clusters termed 'early upregulated'.

Fig. S12. Cellular processes enriched for genes belonging to co-expression clusters termed 'early downregulated'.

Fig. S13. Nuclear genes encoding chloroplast-localised proteins (NGCPs) display synchronised and temporally coordinated expression profiles in response to *Pst* infection.

Fig. S14. Two *TaCSP41a* disruption mutants were selected for functional analysis.

Table S1. Seedling infection assays illustrate the three selected wheat varieties Oakley, Solstice and Santiago have different levels of susceptibility to the two *Pst* isolates F22 and 13/14.

Table S2. Probability of the presence of a mitochondrial, chloroplast and thylakoid luminal transit peptide for TaCSP41a homoeologous proteins.

Table S3. Primers used for RT-qPCR assays and their efficiencies.

Table S4. KASP primers to genotype the *TaCSP41a* disruption TILLING mutant lines.

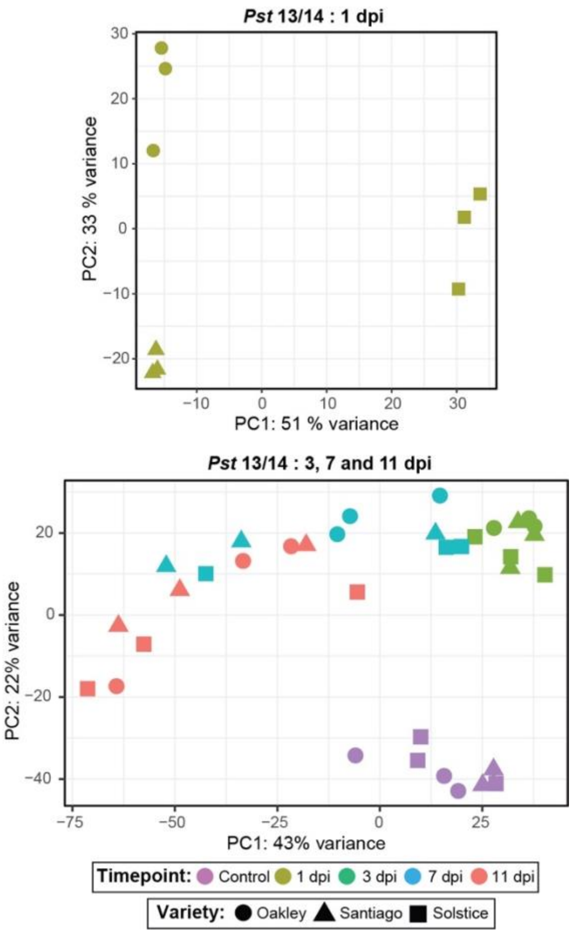

Fig. S1. Principal component (PC) analysis illustrated that samples tended to group at the earliest time-point (1 dpi) by variety following infection with *Pst* isolate 13/14. PC analysis was conducted following separation of samples taken at 1-day post-inoculation (dpi) from all remaining time-points.

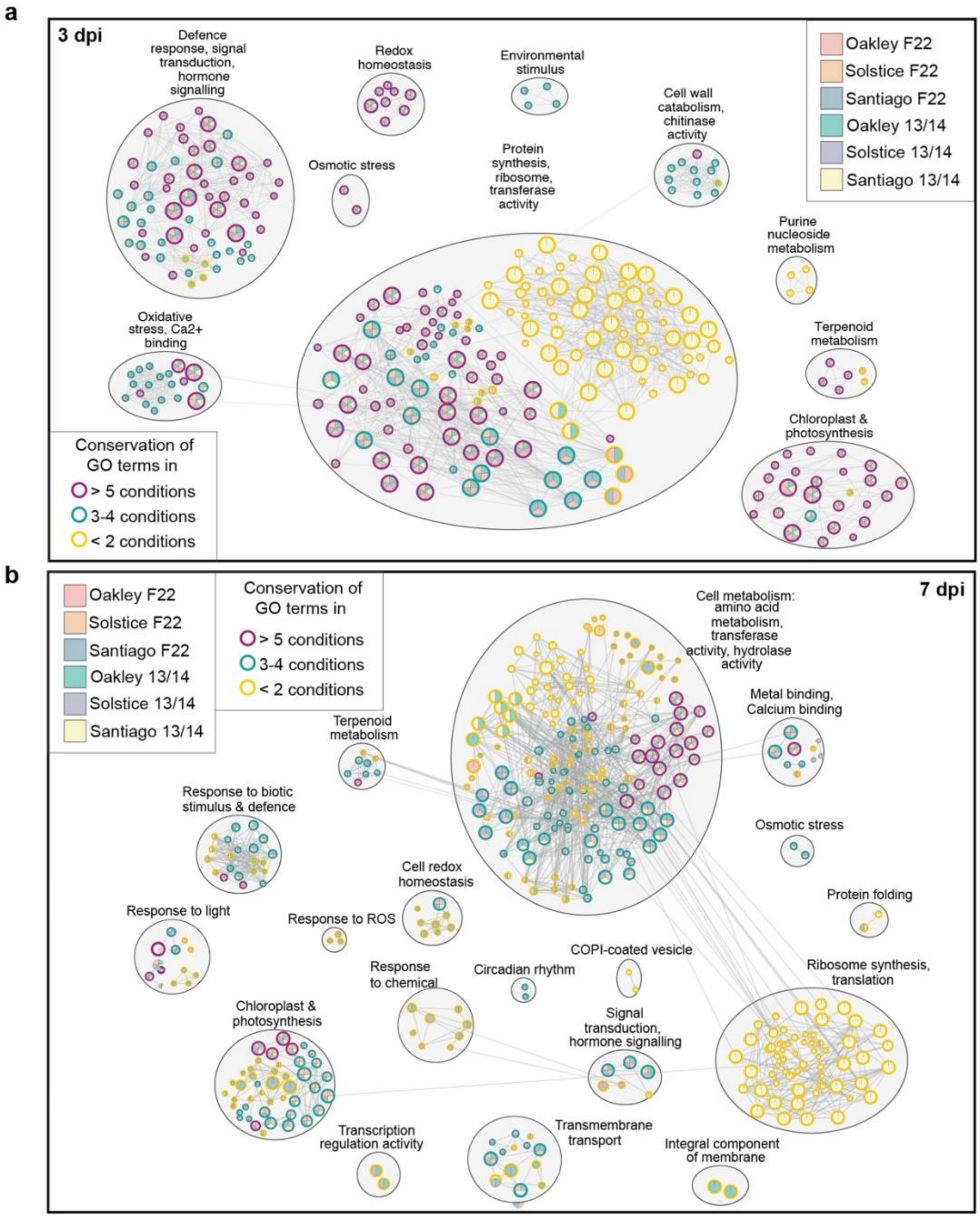

Fig. S2. A number of biological processes were enriched at 3 and 7 days post-inoculation (dpi). Functional enrichment networks for each *Pst*-variety pair were identified in samples taken at 3 **(a)** and 7 **(b)** dpi. Gene ontology (GO) terms were assigned to all differentially expressed genes (DEGs) where possible and those identified as significantly enriched (*q*-value < 0.0005) in at least one *Pst*-varietal pair are represented by a node, with node sizes proportional to the number of genes annotated with the GO term. Edges indicate overlapping member genes and conservation of GO term enrichment is highlighted by node border colour.

Highly similar gene sets formed clusters, which were annotated and labelled with appropriate summarizing terms.

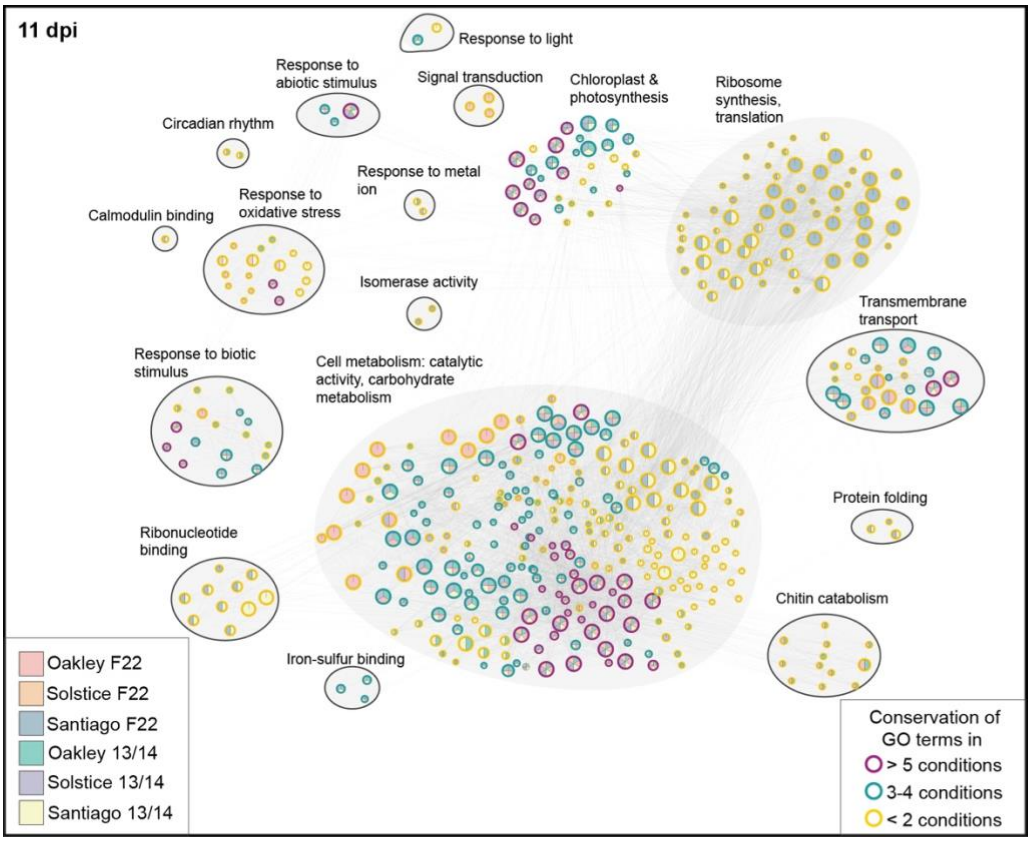

Fig. S3. A number of biological processes were enriched at 11 days post-inoculation (dpi). Functional enrichment networks for each *Pst*-variety pair identified in samples taken at 11 days post-inoculation (dpi). Gene ontology (GO) terms were assigned to all differentially expressed genes (DEGs) where possible and those identified as significantly enriched (q-value < 0.0005) in at least one *Pst*-varietal pair are represented by a node, with node sizes proportional to the number of genes annotated with the GO term. Edges indicate overlapping member genes and conservation of GO term enrichment is highlighted by node border colour. Highly similar gene sets formed clusters, which were annotated and labelled with appropriate summarizing terms.

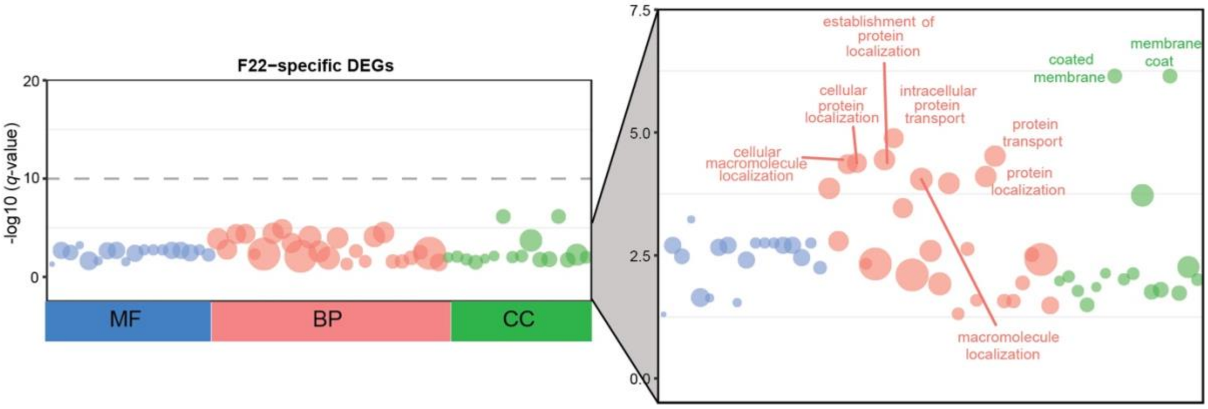

Fig. S4. Gene ontology (GO) terms identified as functional enriched among the 1,494 *Pst* F22-specific DEGs included a large array of biological processes. GO terms were annotated when -log(*q*-value) > 4 and circle size represents the number of genes annotated within the particular enriched function. Circle colour represents the GO term classification: molecular function (MF, blue), biological process (BP, pink) and cellular component (CC, green).

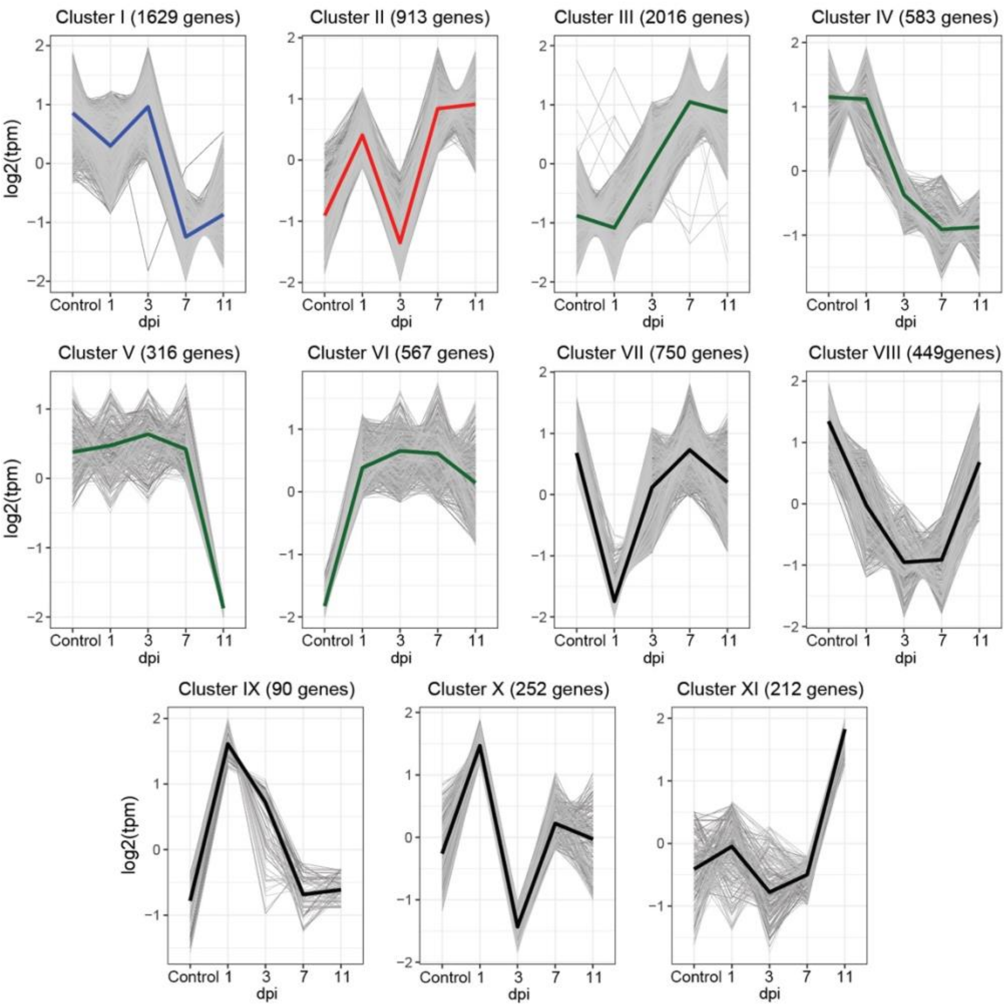

Fig. S5. Eleven co-expression clusters were identified among the 8,627 *Pst* 13/14-specific DEGs following infection of Oakley with *Pst* isolate F22. The coloured line represents the average normalised expression of all genes in a co-expression cluster. Clusters containing genes classified as upregulated or downregulated early during the *Pst* infection process are shown in blue and red respectively. Clusters with global expression differences are shown in dark green and those containing genes where no particular gene ontology (GO) term was identified as enriched are shown in black.

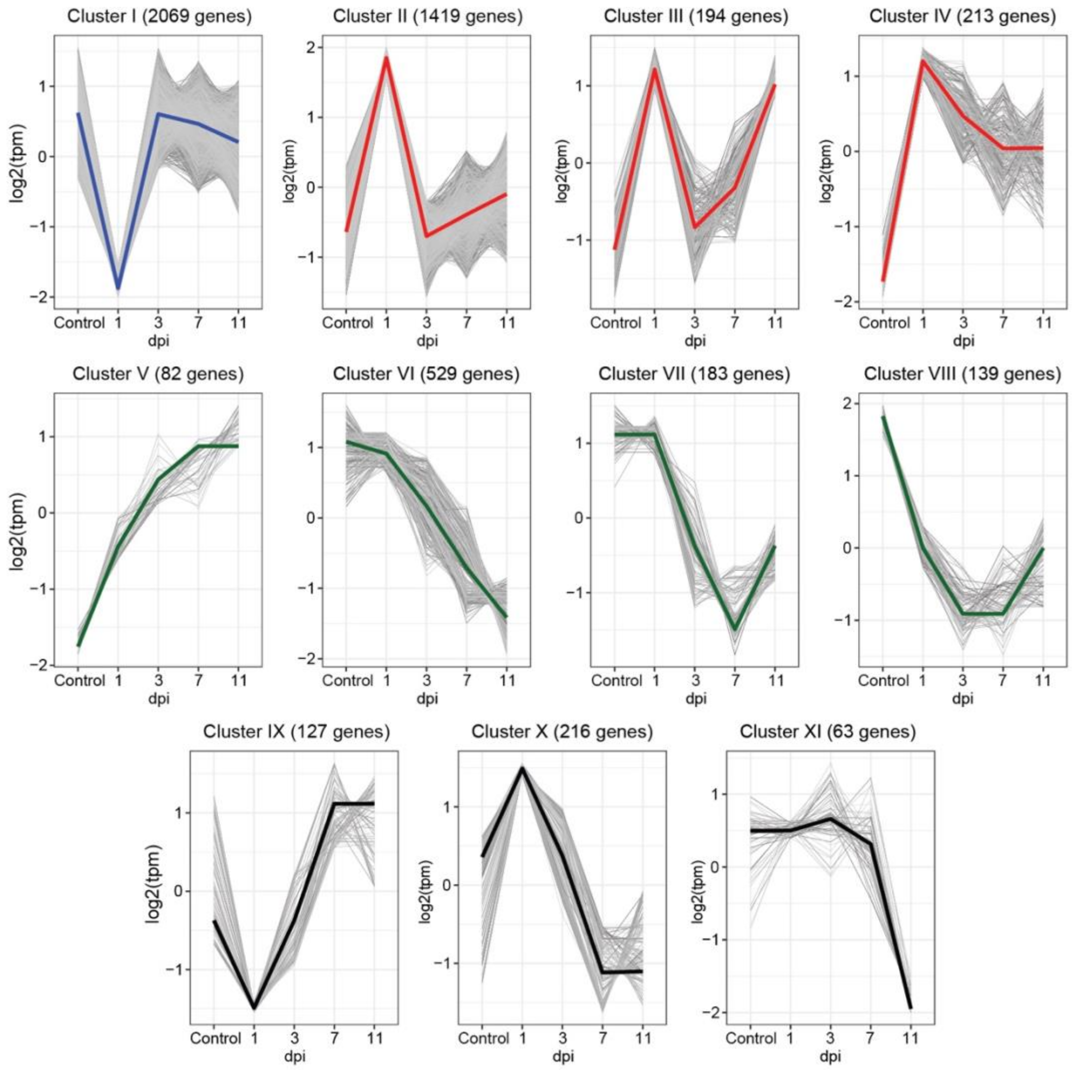

Fig. S6. Eleven co-expression clusters were identified among the 8,627 *Pst* 13/14-specific DEGs following infection of Santiago with *Pst* isolate F22. The coloured line represents the average normalised expression of all genes in a co-expression cluster. Clusters containing genes classified as upregulated or downregulated early during the *Pst* infection process are shown in blue and red respectively. Clusters with global expression differences are shown in dark green and those containing genes where no particular gene ontology (GO) term was identified as enriched are shown in black.

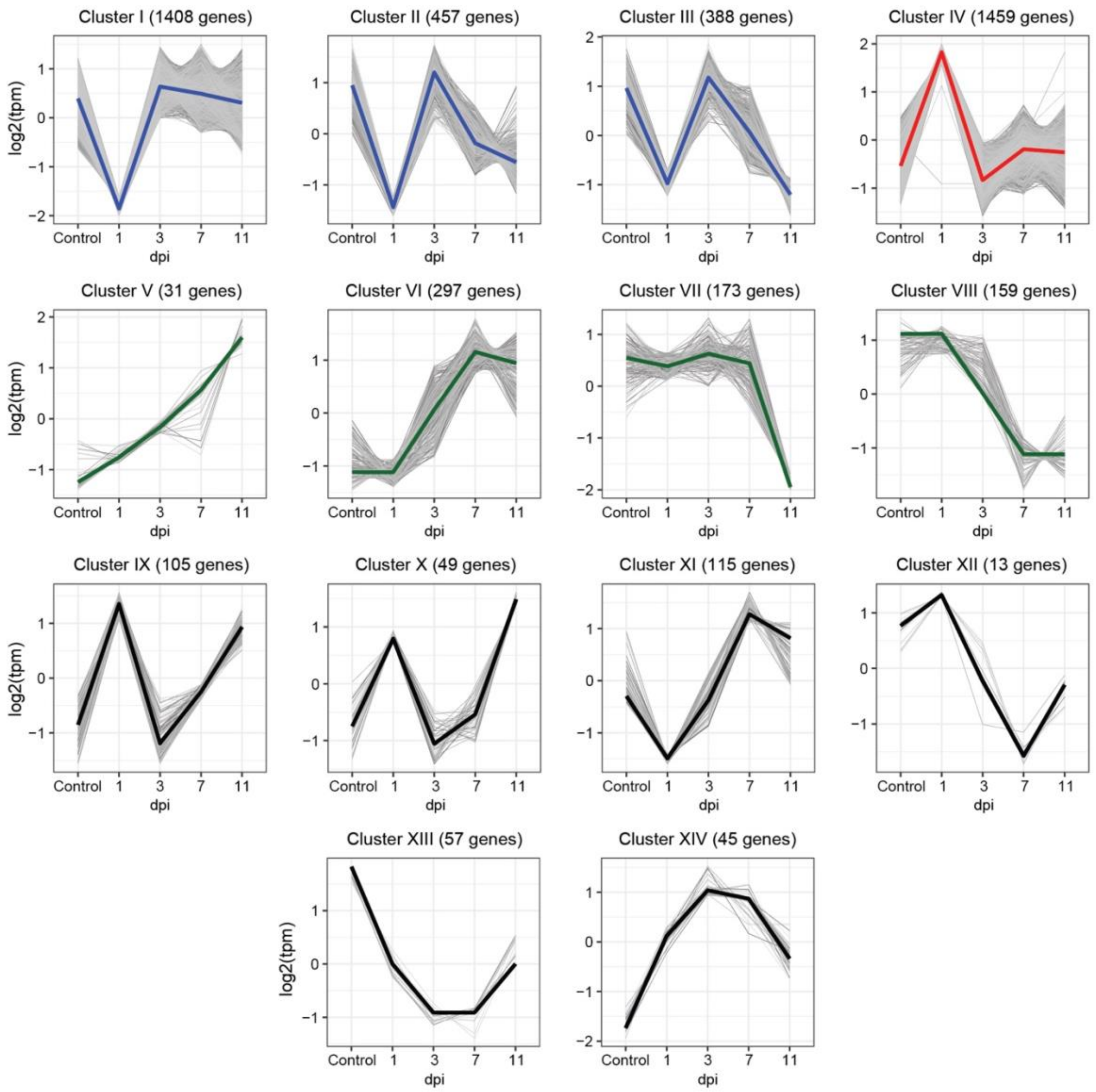

Fig. S7. Fourteen co-expression clusters were identified among the 8,627 *Pst* 13/14-specific DEGs following infection of Solstice with *Pst* isolate F22. The coloured line represents the average normalised expression of all genes in a co-expression cluster. Clusters containing genes classified as upregulated or downregulated early during the *Pst* infection process are shown in blue and red respectively. Clusters with global expression differences are shown in dark green and those containing genes where no particular gene ontology (GO) term was identified as enriched are shown in black.

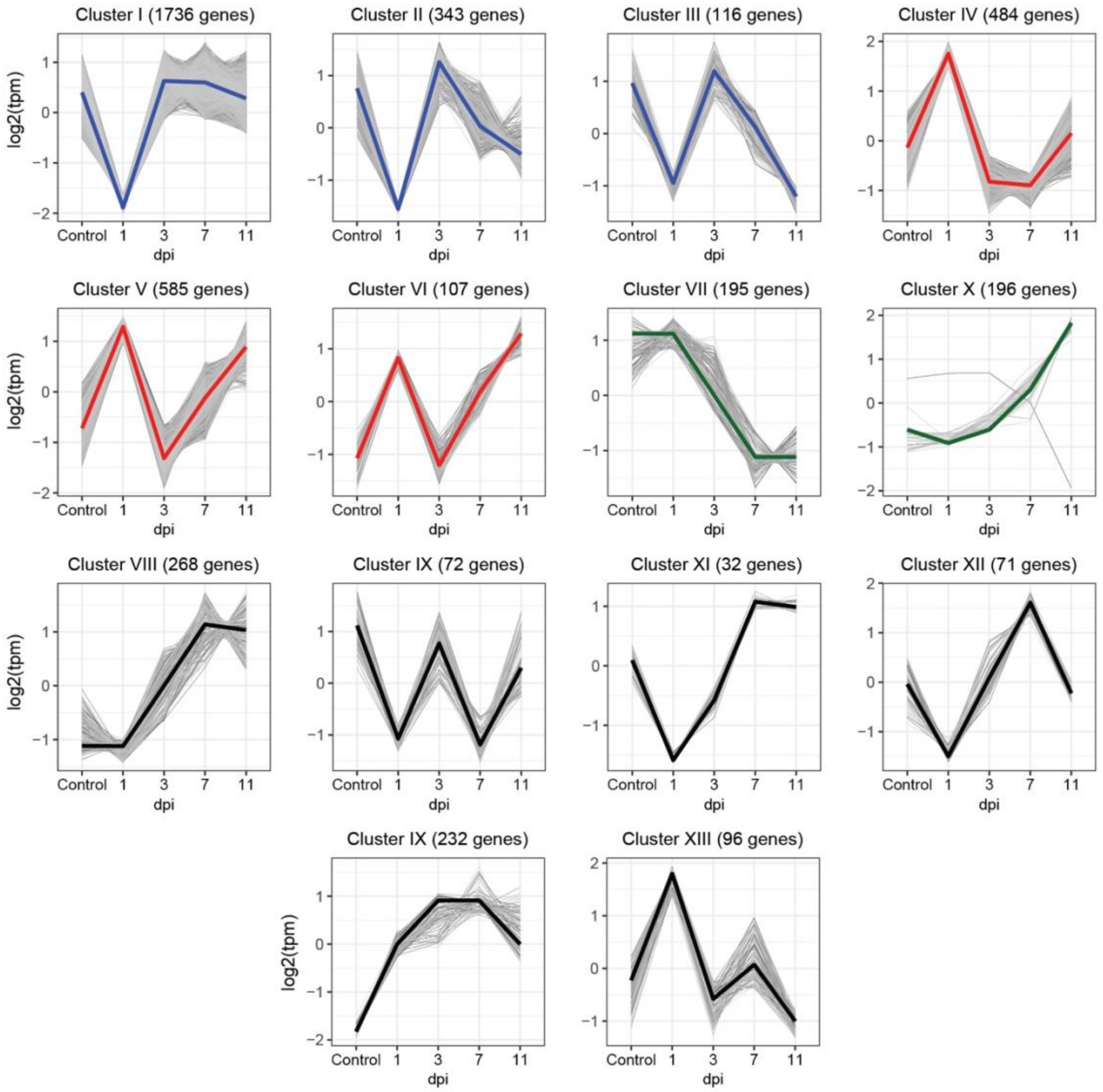

Fig. S8. Fourteen co-expression clusters were identified among the 8,627 *Pst* 13/14-specific DEGs following infection of Oakley with *Pst* isolate 13/14. The coloured line represents the average normalised expression of all genes in a co-expression cluster. Clusters containing genes classified as upregulated or downregulated early during the *Pst* infection process are shown in blue and red respectively. Clusters with global expression differences are shown in dark green and those containing genes where no particular gene ontology (GO) term was identified as enriched are shown in black.

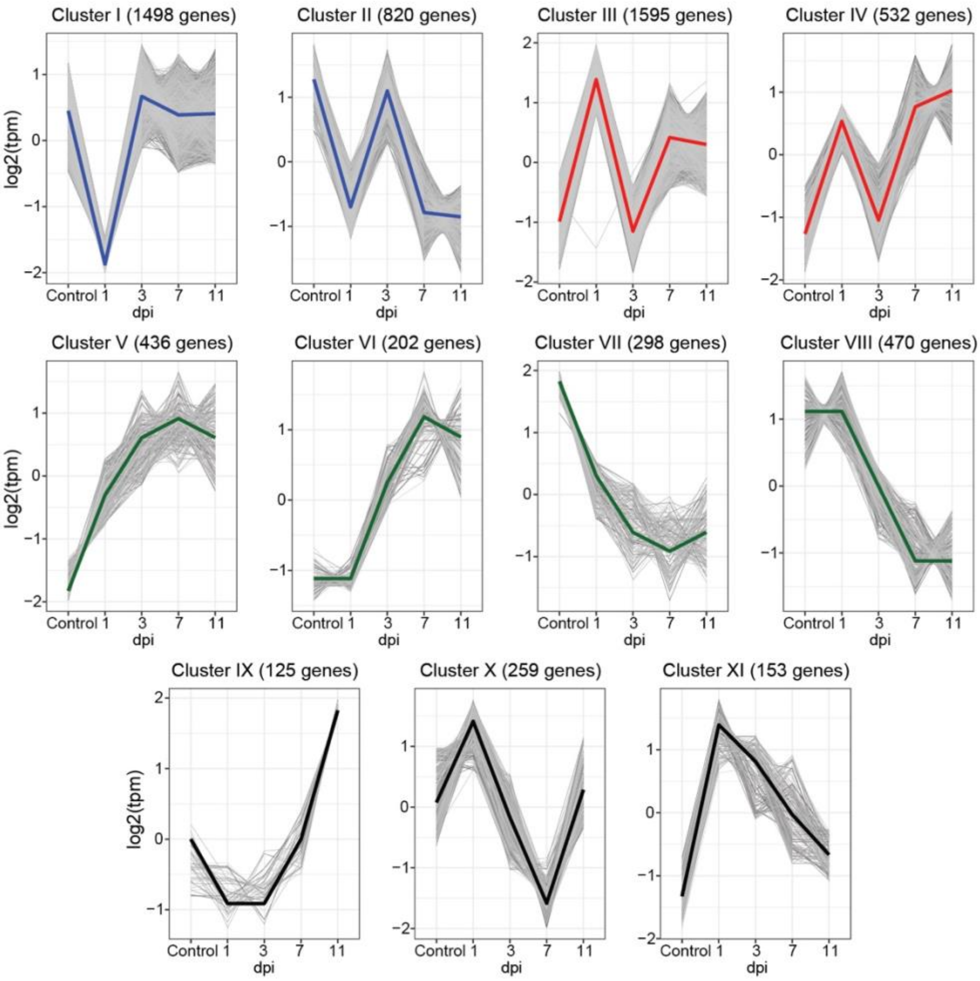

Fig. S9. Eleven co-expression clusters were identified among the 8,627 *Pst* 13/14-specific DEGs following infection of Santiago with *Pst* isolate 13/14. The coloured line represents the average normalised expression of all genes in a co-expression cluster. Clusters containing genes classified as upregulated or downregulated early during the *Pst* infection process are shown in blue and red respectively. Clusters with global expression differences are shown in dark green and those containing genes where no particular gene ontology (GO) term was identified as enriched are shown in black.

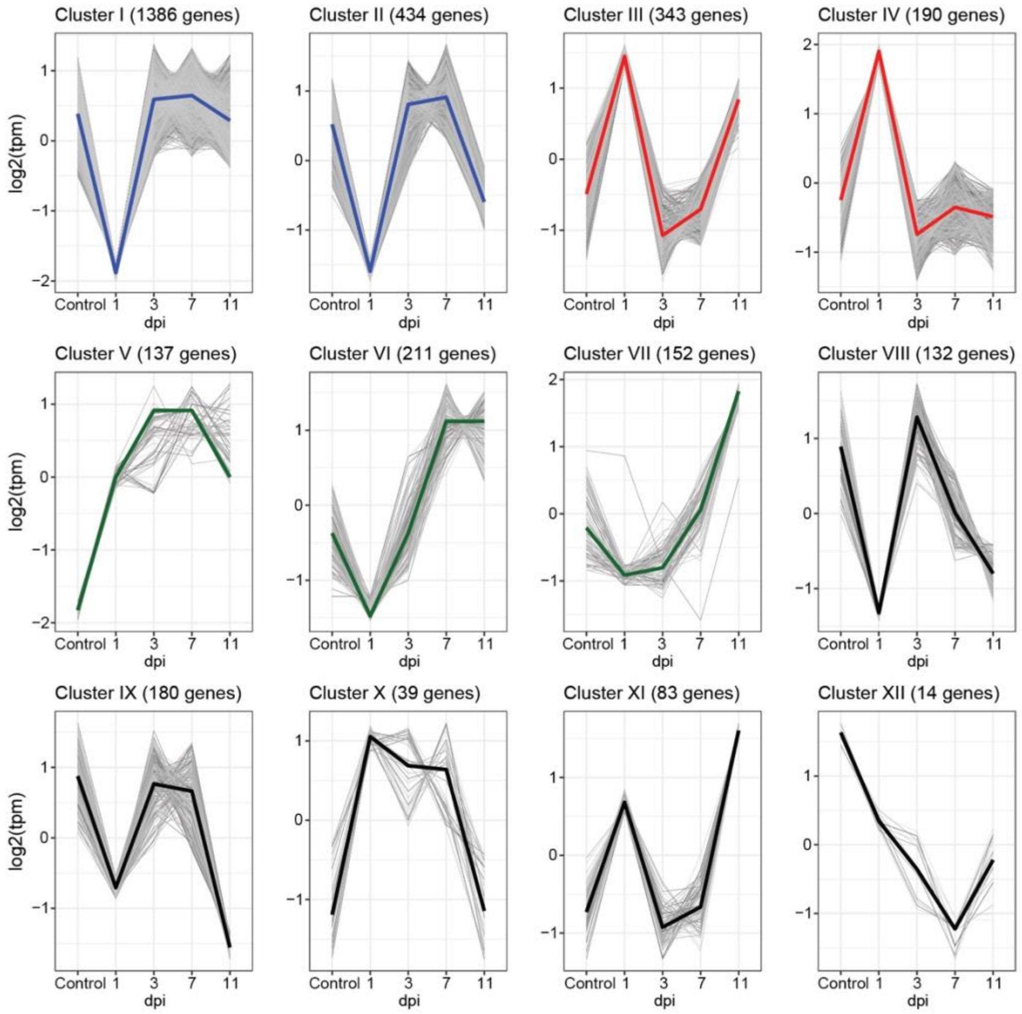

Fig. S10. Twelve co-expression clusters were identified among the 8,627 *Pst* 13/14-specific DEGs following infection of Solstice with *Pst* isolate 13/14. The coloured line represents the average normalised expression of all genes in a co-expression cluster. Clusters containing genes classified as upregulated or downregulated early during the *Pst* infection process are shown in blue and red respectively. Clusters with global expression differences are shown in dark green and those containing genes where no particular gene ontology (GO) term was identified as enriched are shown in black.

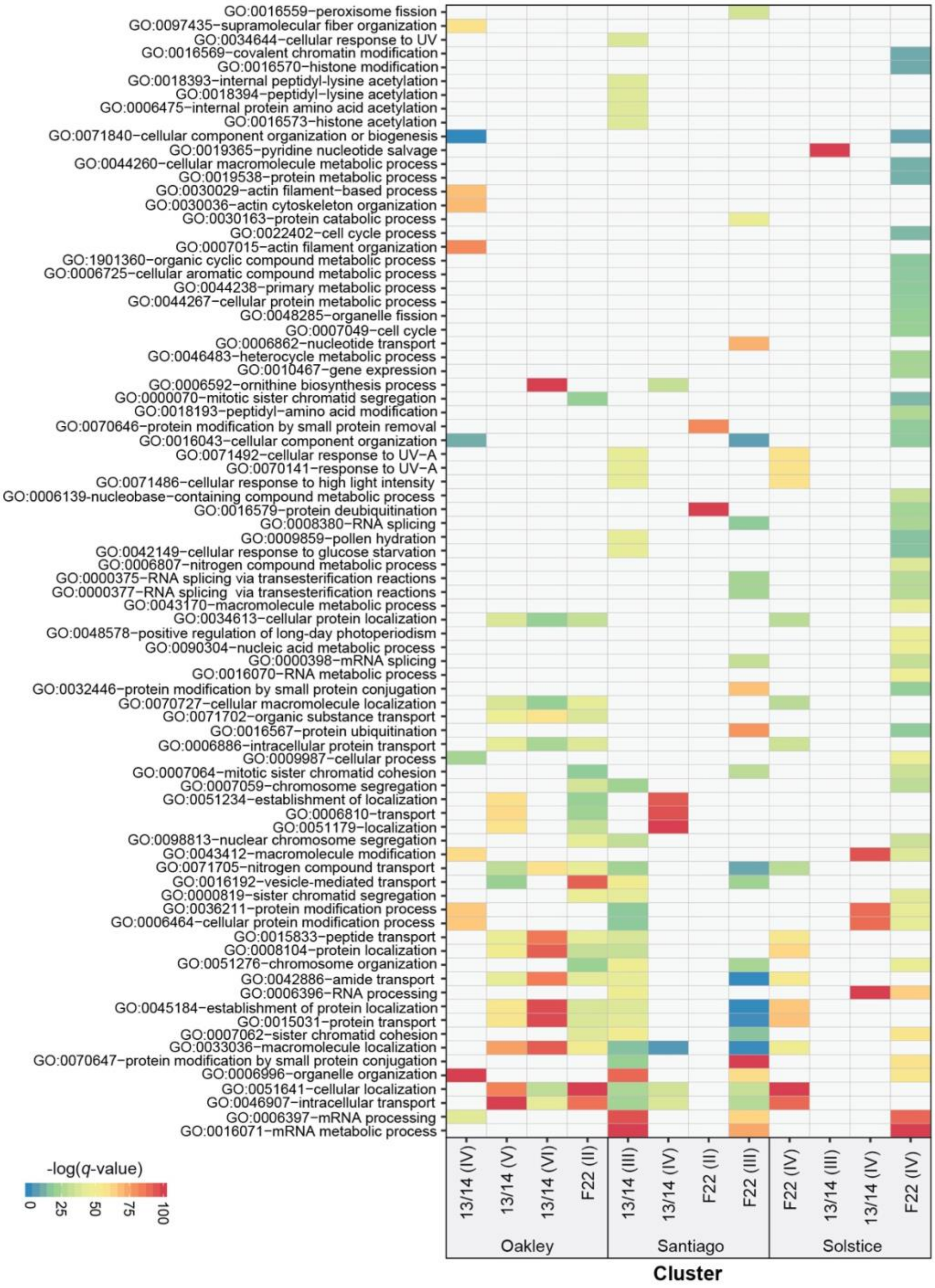

Fig. S11. Cellular processes enriched for genes belonging to co-expression clusters termed ‘early upregulated’. Functional enrichment analysis was undertaken using genes that belonged to co-expression clusters that were built utilising genes from all *Pst*-varietal pairs that displayed high levels of expression at 1-day post-inoculation, among the 8,627 *Pst* 13/14-specific differentially expressed genes (DEGs). Significant -log(*q*-value) are represented in a 0-100 scale (blue to red) and gene ontology (GO) terms with -log(*q*-value) > 2 are presented.

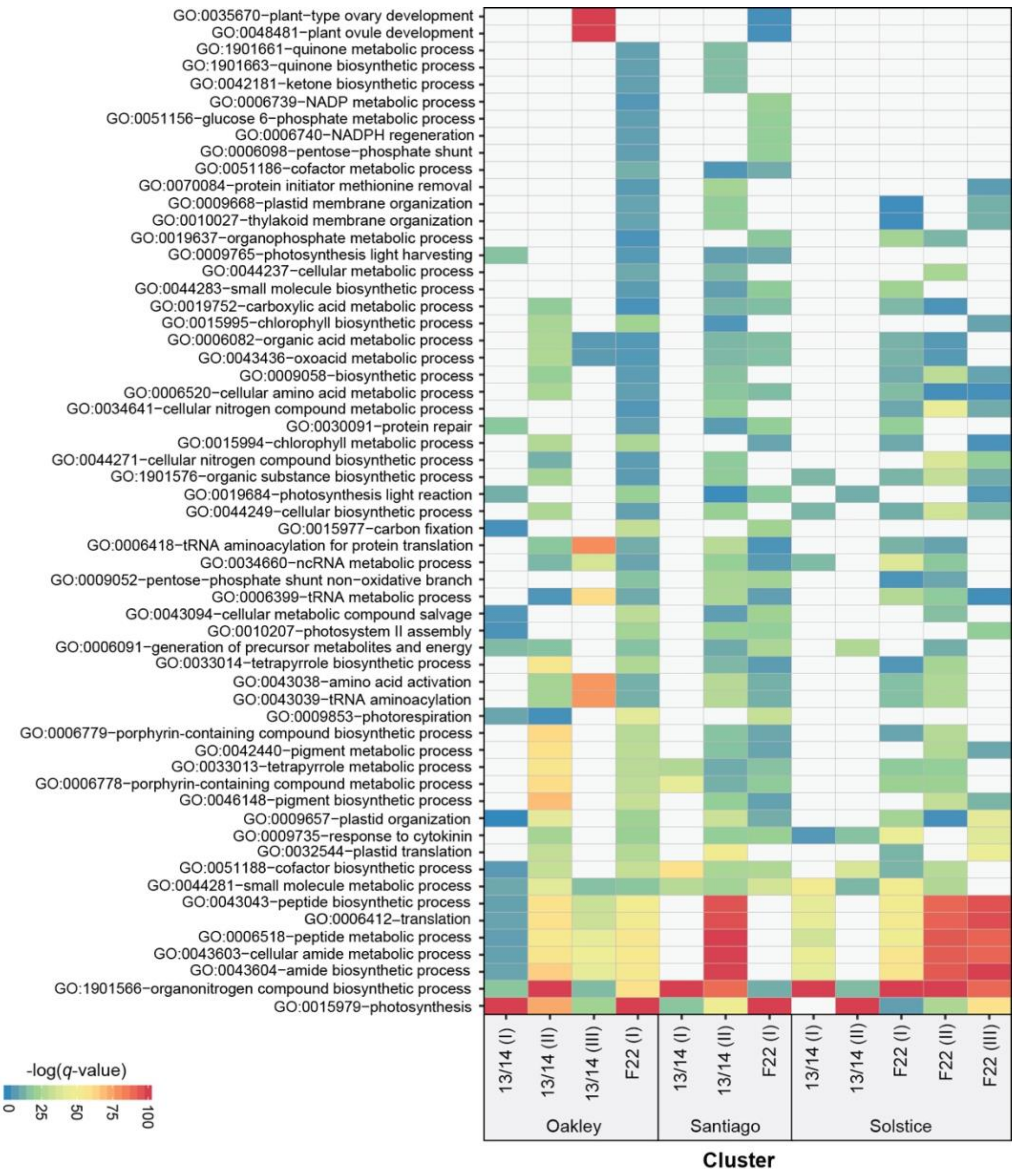

Fig. S12. Cellular processes enriched for genes belonging to co-expression clusters termed 'early downregulated'. Functional enrichment analysis was undertaken using genes that belonged to co-expression clusters that were built utilising genes from all *Pst*-varietal pairs and that displayed low levels of expression at 1-day post-inoculation, among the 8,627 *Pst* 13/14-specific differentially expressed genes (DEGs). Significant -log(*q*-value) values are represented using a 0-100 scale and gene ontology (GO) terms with -log(*q*-value) > 5 are presented.

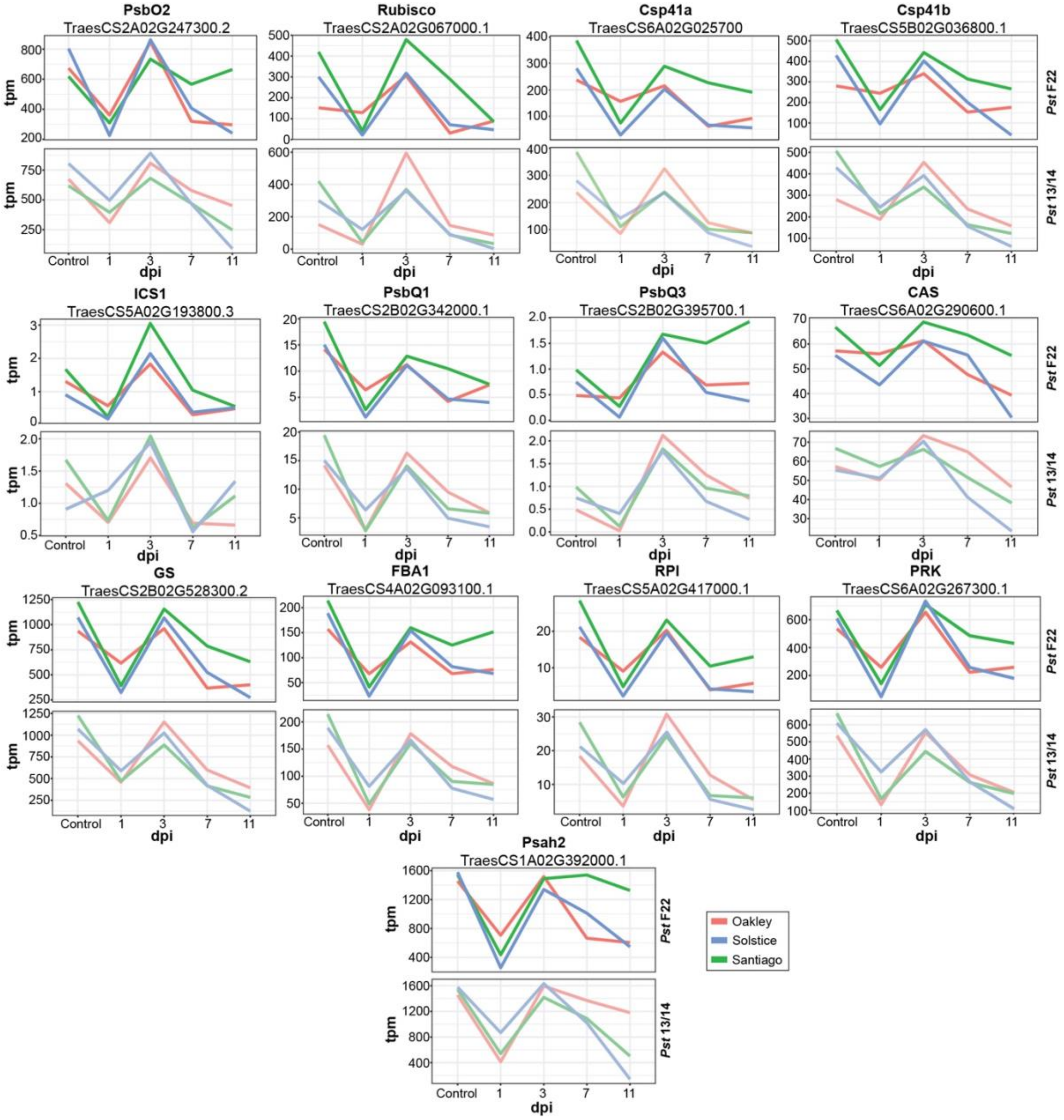

Fig. S13. Nuclear genes encoding chloroplast-localised proteins (NGCPs) display synchronised and temporally coordinated expression profiles in response to *Pst* infection. Graphs represent median expression values of normalised transcripts per million (tpm) values obtained for a subset of genes predicted to encode proteins with functions in the chloroplast.

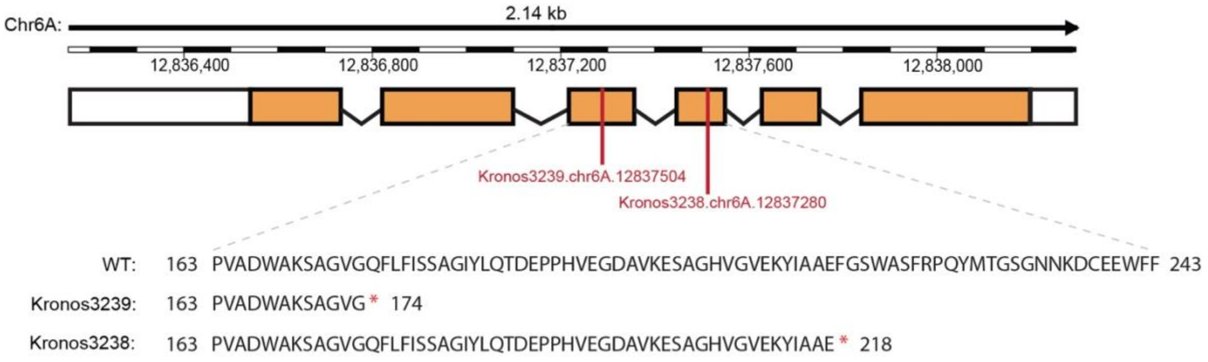

Fig. S14. Two *TaCSP41a* disruption mutants were selected for functional analysis. *TaCSP41a* is located on chromosome 6 and two loss-of-function tetraploid Kronos targeting in local lesions in genomes (TILLING) mutants were identified in the A genome. The two *TaCSP41a* disruption mutants induced early stop codon mutations independently at amino acids 218 or 174. Chr, chromosome; kb, kilobase; WT, wild type.

Table S1. Seedling infection assays illustrate that the three selected wheat varieties Oakley, Solstice and Santiago have different levels of susceptibility to the two *Pst* isolates F22 and 13/14. Each wheat variety was subjected to *Pst* infection with the two isolates (F22 and 13/14) and infection types (IT) recorded 12 days post-inoculation following the 0–4 scale[1]. Between 7 to 9 independent plants considered as biological replicates were analysed. n.d., not determined.

		**Plant replicate**										
***Pst* isolate**	**Wheat variety**	**1**	**2**	**3**	**4**	**5**	**6**	**7**	**8**	**9**	**Average infection type**	**Host response**
F22	Solstice	2	2	3	3	3	3	2	2	3	2.6	Moderate susceptibility
F22	Santiago	0	1	1	1	2	1	1	1	0	0.9	Resistant
F22	Oakley	3	4	4	3	3	3	3	4	3	3.3	Full susceptibility
13/14	Solstice	3	4	4	2	3	3	2	3	4	3.1	Full susceptibility
13/14	Santiago	2	2	2	2	2	2	4	n.d.	n.d.	2.3	Moderate resistance
13/14	Oakley	3	4	3	3	3	4	4	4	4	3.6	Full susceptibility

Table S2. Probability of the presence of a mitochondrial, chloroplast and thylakoid luminal transit peptide for TaCSP41a homoeologous proteins. The program TargetP (version 2.0) was used to search the protein sequences for signatures typical of a chloroplast transit peptide. Values represent percentage probability.

Homoeolog	Mitochondrial transit peptide	Chloroplast transit peptide	Thylakoid luminal transit peptide
**TraesCS6A02G025700**	0.0002	0.9984	0.0013
**TraesCS6B02G036400**	0.0001	0.9983	0.0014
**TraesCS6D02G029300**	0.0002	0.9985	0.0012

Table S3. Primers used for RT-qPCR assays and their efficiencies. Primers were designed to quantify the expression of all three homoeologous copies of each gene simultaneously.

**Gene name**	**Gene ID Refseq v1.1**	**Forward primer**	**Reverse primer**	**Efficiency**
TaCSP41a (common)	TraesCS6A02G025700, TraesCS6B02G036400, TraesCS6D02G029300	TTTCGAACGTGTGAGCCTGA	TCACAACGGATTGGCGATGA	98.70%
UBC4 (Reference gene)[2]	TraesCS4A02G414200, TraesCS4B02G314900, TraesCS4D02G312000	ACAAGGTCGAGACGGTGAAC	GTAAGGATACGCATCGGGCA	104.31%

Table S4. KASP primers to genotype the *TaCSP41a* disruption TILLING mutant lines. Primers were designed with additional standard FAM or HEX compatible tails. WT primers (A) were designed with FAM tails (5′ GAAGGTGACCAAGTTCATGCT 3′) and MUT primers (B) with HEX tails (5′ GAAGGTCGGAGTCAACGGATT 3′).

	Kronos TILLING lines	
**Primer**	**3238**	**3239**
WT primer (A)	gcGgagttcggcagctgG	gctgctgatgaacaggaactG
MUT primer (B)	gcGgagttcggcagctgA	gctgctgatgaacaggaactA
Common primer	tcgaggctgaatgattaagtgatta	AgttttgatGtttggttgcgT

References:

1 McIntosh, R. A., Wellings, C. R. & Park, R. F. Wheat rusts: an atlas of resistance genes. (CSIRO Publications, 1995).

2 Borrill, P., Ramirez-Gonzalez, R. & Uauy, C. expVIP: a Customizable RNA-seq Data Analysis and Visualization Platform. *Plant Physiol* **170**, 2172-2186, doi:10.1104/pp.15.01667 (2016).
