# Supplementary material for: Temporally coordinated expression of nuclear genes encoding chloroplast proteins in wheat promotes Puccinia striiformis f. sp. tritici infection
Source: Commun Biol. 2022 Aug 22;5:853. doi: 10.1038/s42003-022-03780-4 (PMC9395331; doi:10.1038/s42003-022-03780-4)
Supplement: Supplementary file 2 — Description of Additional Supplementary Files [file 42003_2022_3780_MOESM2_ESM.pdf]

Description of Additional Supplementary Files

File name: Supplementary Data 1
Description: Transcripts per million (tpm) values for the 1,494 differentially expressed genes (DEGs) specifically identified during infecton of the three wheat varieties (Oakley, Santiago and Solstice) with *Pst* isolate F22. Rep, independent biological replicate (independant *Pst*-infected plants).

File name: Supplementary Data 2
Description: Transcripts per million (tpm) values for the 8,627 differentially expressed genes (DEGs) specifically identified during infecton of the three wheat varieties (Oakley, Santiago and Solstice) with *Pst* isolate 13/14. Rep, independent biological replicate (independant *Pst*-infected plants).

File name: Supplementary Data 3
Description: Eight second level gene ontology (GO) terms with chloroplast-related functions were significantly enriched in one or more co-expression clusters.

File name: Supplementary Data 4
Description: All raw data measurements to support the analyses presented.
