# Supplementary material for: Temporally coordinated expression of nuclear genes encoding chloroplast proteins in wheat promotes Puccinia striiformis f. sp. tritici infection
Source: Commun Biol. 2022 Aug 22;5:853. doi: 10.1038/s42003-022-03780-4 (PMC9395331; doi:10.1038/s42003-022-03780-4)
Supplement: Supplementary file 7 — Reporting Summary [file 42003_2022_3780_MOESM7_ESM.pdf]

nature portfolio

Corresponding author(s): Diane G.O. Saunders

Last updated by author(s): Jun 30, 2022

Reporting Summary

Nature Portfolio wishes to improve the reproducibility of the work that we publish. This form provides structure for consistency and transparency in reporting. For further information on Nature Portfolio policies, see our Editorial Policies and the Editorial Policy Checklist.

Statistics

For all statistical analyses, confirm that the following items are present in the figure legend, table legend, main text, or Methods section.

n/a	Confirmed	
☐	☒	The exact sample size (*n*) for each experimental group/condition, given as a discrete number and unit of measurement
☐	☒	A statement on whether measurements were taken from distinct samples or whether the same sample was measured repeatedly
☐	☒	The statistical test(s) used AND whether they are one- or two-sided<br>*Only common tests should be described solely by name; describe more complex techniques in the Methods section.*
☒	☐	A description of all covariates tested
☐	☒	A description of any assumptions or corrections, such as tests of normality and adjustment for multiple comparisons
☐	☒	A full description of the statistical parameters including central tendency (e.g. means) or other basic estimates (e.g. regression coefficient) AND variation (e.g. standard deviation) or associated estimates of uncertainty (e.g. confidence intervals)
☐	☒	For null hypothesis testing, the test statistic (e.g. *F*, *t*, *r*) with confidence intervals, effect sizes, degrees of freedom and *P* value noted<br>*Give P values as exact values whenever suitable.*
☒	☐	For Bayesian analysis, information on the choice of priors and Markov chain Monte Carlo settings
☒	☐	For hierarchical and complex designs, identification of the appropriate level for tests and full reporting of outcomes
☒	☐	Estimates of effect sizes (e.g. Cohen's *d*, Pearson's *r*), indicating how they were calculated

Our web collection on statistics for biologists contains articles on many of the points above.

Software and code

Policy information about availability of computer code

Data collection

Data used in this study was generated by the authors.

Data analysis

Filtering of RNA-seq data: RNA samples were sent to GENEWIZ (UK) for cDNA library preparation using an Illumina TruSeq RNA Sample Preparation Kit (Illumina, US) and sequencing on an Illumina HiSeq 2500 instrument. For pseudoalignment to the wheat reference transciptome, the resulting 150-bp paired-end reads were quality-trimmed and filtered using the package fastp v.0.12.3 (Chen et. al. 2018) with default parameters (Phred quality >=15; percent unqualified bases = 40; low complexity filter = 30%; adaptor trimmed by overlap analysis). Alternatively, raw reads from each sample were independently filtered using BBDUK from BBTools version 37.68 and aligned to the Pst reference genome (isolate Pst-104E) using STAR version 2.5.a.

Transcript quantification and differential gene expression analysis: Transcript abundance was assessed for each of the 81 Pst-infected and mock-inoculated Oakley, Solstice and Santiago samples following pseudoalignment to the wheat reference transcriptome (RefSeq v1.1 from the cultivar Chinese Spring) using kallisto version 0.43.0 (Bray et al., 2016). Reads from each sample were independently aligned to the Pst reference genome (isolate PST-130) as described previously (Hubbard et al., 2015). Transcript per million (tpm) counts were imported into R using tximport version 1.8.0. Principal Component Analysis (PCA) and differential gene expression analysis were performed using normalised counts in the R package DESeq2 version v.1.16.1 (Love et al., 2014). Pairwise comparisons were performed for each Pst-infected sample against its appropriate mock-inoculated sample and Log2 Fold-Change (LFC) values and Benjamini and Hochberg-adjusted p-values were determined. Genes were considered to be differentially expressed when adjusted p-values (q-value) < 0.05.

Gene ontology (GO) term enrichment analysis and co-expression clustering: GO term enrichment analysis was performed as described previously (Reimand et al., 2019). In brief, the g:GOSt tool in g:Profiler (Raudvere et al., 2019) was used with the g:SCS algorithm and a p-value cutoff for overrepresentation of < 0.05. For enrichment analysis at each time point for individual cultivar-Pst isolate pairs, visualisation of GO term networks was performed with the Cytoscape (v3.7) Enrichment Map plug-in (v3.2.1) using a node false discovery rate (FDR) q-value cutoff of <0.0005. Clusters of gene sets were annotated according to their GO term annotation using Cytoscape Autoannotate (v1.3.2) and

Word Cloud (v3.1) plug-ins. Gene lists for specific GO terms or genes carrying specific domains were obtained using EnsemblPlants BioMart (Smedley et al., 2015) with wheat gene annotation (RefSeq v1.1). A total of 8,627 differentially expressed genes (DEGs) were identified at 1 dpi that were specific to infection with Pst isolate 13/14, whose Log2-normalised tpm values were used to build co-expression clusters using Clust (Abu-Jamous et al., 2018) with a tightness level of 0.5 (-t 0.5).

Annotation of TaCSP41a: The predicted subcellular localisation of TaCSP41a encoded by the three homoeologues (TraesCS6A02G025700, TraesCS6B02G036400 and TraesCS6D02G029300) was assessed using TargetP (Emanuelsson et al., 2007) using default parameters (Supplementary Table S2).

For manuscripts utilizing custom algorithms or software that are central to the research but not yet described in published literature, software must be made available to editors and reviewers. We strongly encourage code deposition in a community repository (e.g. GitHub). See the Nature Portfolio guidelines for submitting code & software for further information.

Data

Policy information about availability of data

All manuscripts must include a data availability statement. This statement should provide the following information, where applicable:

- Accession codes, unique identifiers, or web links for publicly available datasets
- A description of any restrictions on data availability
- For clinical datasets or third party data, please ensure that the statement adheres to our policy

Sequence data that support the findings of this study can be found in the European Nucleotide Archive (ENA) database under the following accession number: PRJEB50522.

Field-specific reporting

Please select the one below that is the best fit for your research. If you are not sure, read the appropriate sections before making your selection.

☒ Life sciences ☐ Behavioural & social sciences ☐ Ecological, evolutionary & environmental sciences

For a reference copy of the document with all sections, see nature.com/documents/nr-reporting-summary-flat.pdf

Life sciences study design

All studies must disclose on these points even when the disclosure is negative.

Sample size	RNA-seq data: Three biological replicates (three different plants) were collected per time point (1, 3, 7 and 11 dpi) for each wheat variety-Pst isolate pair.
Data exclusions	No data was excluded.
Replication	Subcellular localisation of TaCSP41a: Images are representative of > 10 images captured, all displaying co-localisation of TaCSP41a-A-GFP and chlorophyll autofluorescence.
Randomization	Randomization was not relevant to this study.
Blinding	Blinding was not relevant to this study.

Reporting for specific materials, systems and methods

We require information from authors about some types of materials, experimental systems and methods used in many studies. Here, indicate whether each material, system or method listed is relevant to your study. If you are not sure if a list item applies to your research, read the appropriate section before selecting a response.

Materials & experimental systems

n/a	Involved in the study	
☒	☐	Antibodies
☒	☐	Eukaryotic cell lines
☒	☐	Palaeontology and archaeology
☒	☐	Animals and other organisms
☒	☐	Human research participants
☒	☐	Clinical data
☒	☐	Dual use research of concern

Methods

n/a	Involved in the study	
☒	☐	ChIP-seq
☒	☐	Flow cytometry
☒	☐	MRI-based neuroimaging
